# *Kcnq2* R213 knock-in mice reveal variant- and region-specific mechanisms underlying self-limited familial neonatal-infantile epilepsy and early infantile developmental and epileptic encephalopathy

**DOI:** 10.1186/s40478-026-02264-4

**Published:** 2026-02-25

**Authors:** Takuma Nishijo, Nanako Hamada, Reut Suliman-Lavie, Hidenori Tabata, Hidenori Ito, Ikuko Iwamoto, Sagiv Shifman, Koh-ichi Nagata

**Affiliations:** 1 Department of Molecular Neurobiology, Institute for Developmental Research, Aichi Developmental Disability Center, 713-8 Kamiya, Kasugai, Aichi 480-0392 Japan; 2 Department of Genetics, The Alexander Silberman Institute of Life Sciences The Hebrew University of Jerusalem, 91904 Jerusalem, Israel; 3 Department of Neurochemistry, Nagoya University Graduate School of Medicine, 65 Tsurumai-Cho, Showa-Ku, Nagoya, Aichi 466-8550 Japan; 4 Present Address: Laboratory of Physical Chemistry, Faculty of Pharmaceutical Sciences, Teikyo University, Itabashi-ku, Tokyo, 173-8605 Japan

**Keywords:** KCNQ2, Self-limited familial neonatal-infantile epilepsy, Early infantile developmental epileptic encephalopathy, Pathogenic variants, Knock-in mice

## Abstract

*KCNQ2* variants cause a spectrum of neonatal epilepsies, ranging from self-limited familial neonatal-infantile epilepsy (SeLFNIE) to early infantile developmental and epileptic encephalopathy (EIDEE). Two distinct missense variants at the same residue, p.R213W and p.R213Q, are associated with SeLFNIE and EIDEE, respectively. This study aimed to elucidate the in vivo effects of these variants on brain development and neuronal excitability using two knock-in mouse models, *Kcnq2*^R213W/+^ and *Kcnq2*^R213Q/+^. We assessed survival, seizure susceptibility, histological and molecular phenotypes, and electrophysiological properties in cortical and hippocampal neurons, and conducted RNA sequencing analyses of cortical tissue to identify transcriptional alterations. *Kcnq2*^R213Q/+^ mice exhibited tonic–clonic seizures, shortened lifespan, delayed cortical neuron migration, abnormal elongation of the axon initial segment in cortical neurons, and dentate gyrus-specific gliosis. In contrast, *Kcnq2*^R213W/+^ mice showed a milder phenotype with transient seizures and largely preserved cortical function. RNA sequencing analyses revealed that p.R213Q selectively upregulated genes involved in endoplasmic reticulum stress and synaptic regulation, together with compensatory upregulation of potassium channel subunits. These findings demonstrate that the two *Kcnq2* variants lead to distinct neurodevelopmental phenotypes, attributable not only to differential impairment of the M-current but also to aberrant cortical development and stress response pathways. In particular, p.R213Q induces sustained cortical hyperexcitability and axon initial segment abnormalities, whereas p.R213W results in the milder phenotype. The established knock-in models provide powerful tools for elucidating disease mechanisms of EIDEE and SeLFNIE, and developing targeted therapies for *KCNQ2*-related epilepsies.

**Supplementary Information:**

The online version contains supplementary material available at 10.1186/s40478-026-02264-4.

## Introduction

*KCNQ2* encodes the Kv7.2 subunit of the voltage-gated potassium channel, which pairs with Kv7.3 (encoded by *KCNQ3*) to form heterotetrameric channels generating the M-current (I_M_). This non-inactivating K^+^ current stabilizes the neuronal membrane potential and regulates action potential generation by controlling its threshold [1]. Kv7.2 plays a crucial role in regulating neuronal excitability, and its importance in brain development is highlighted by neonatal-onset epilepsy caused by *KCNQ2* variants, spanning a clinical spectrum from benign self-limited familial neonatal-infantile epilepsy (SeLFNIE) to severe early infantile developmental and epileptic encephalopathy (EIDEE), which is characterized by severe seizures and neurocognitive delay [2, 3].

Haploinsufficiency caused by the loss-of-function of a single *KCNQ2* allele, typically due to nonsense, splice-site, or frameshift variants, is the most common genetic mechanism underlying SeLFNIE, accounting for over 90% of molecularly confirmed cases [4]. In contrast, de novo missense variants are frequently associated with EIDEE, a more severe epileptic phenotype [5–9]. Variants causing complete loss of function or exerting dominant-negative effects tend to result in EIDEE, whereas milder functional reductions are linked to SeLFNIE [10]. Notably, missense variants associated with both SeLFNIE and EIDEE are distributed broadly across the Kv7.2 protein – including the voltage-sensing domain (VSD), pore region, and cytoplasmic domains – without a clear correlation to clinical severity [11]. This suggests that a variant’s functional and phenotypic outcome is not determined solely by its location in the protein. Rather, the biochemical nature of the amino acid substitution and its effect on channel gating or assembly (perhaps in a development- or cell-specific context) likely drive the clinical severity. Although accumulating evidence shows that many pathogenic variants reduce I_M_ and thereby increase neuronal excitability, the precise pathophysiological significance of *KCNQ2* variants in SeLFNIE and EIDEE, as well as the molecular mechanisms underlying their functional defects, remain to be fully elucidated.

It is noteworthy that two distinct de novo heterozygous missense variants at residue R213 of Kv7.2, a positively charged site within the S4 transmembrane segment of the VSD, have been identified in children with divergent clinical outcomes. The variant (c.637C > T, p.R213W) is associated with SeLFNIE [12], while the variant (c.638G > A, p.R213Q) is linked to EIDEE [5, 13–15]. In vitro electrophysiological studies suggest that the significant phenotypic differences between these two variants are related to the degree of functional impairment: both variants reduce channel voltage sensitivity, but p.R213Q produces more severe impairment, potentially underlying its drastic clinical presentation [5]. In silico structural analyses further suggest that substitution with tryptophan (p.R213W), but not glutamine (p.R213Q), stabilizes the VSD through a unique aromatic interaction [5], implying that different amino acid substitutions at R213 exert distinct effects on Kv7.2 function. Nevertheless, it remains unclear whether the substantial clinical divergence can be fully explained by differences in I_M_ impairment. Other, yet unidentified mechanisms may also contribute to the severe phenotype observed with the p.R213Q variant. Supporting this, a pathological analysis of a post-mortem brain from a patient with EIDEE carrying the p.G215R variant revealed cortical malformations, including the presence of heterotopic neurons in deep white matter, when compared to an age-matched control [16]. These findings emphasize the critical role of Kv7.2 in cortical development. However, both the pathophysiological significance of these variants and the precise molecular mechanisms by which they influence cortical architecture remain to be elucidated.

In this study, we generated two knock-in (KI) mouse models, *Kcnq2*^R213W/+^ for SeLFNIE and *Kcnq2*^R213Q/+^ for EIDEE. Analyses of these models revealed structural and functional neuronal changes associated with each variant, indicating region- and variant-specific effects on neuronal excitability. These findings provide new insights into the mechanisms driving the contrasting phenotypes of benign and severe *KCNQ2*-related epilepsies.

## Material and methods

### Plasmids

Mouse *Kcnq2* cDNA was kindly provided by Prof. Naoto Hoshi (University of California, Irvine), and subcloned into the pCAG-Myc vector (Addgene Inc., Cambridge, MA). Using pCAG-Myc-*Kcnq2* as a template, two variants, c.637C > T (p.R213W) and c.638G > A (p.R213Q), were introduced using KOD-Plus Mutagenesis kit (Toyobo, Osaka, Japan). All constructs were verified by DNA sequencing. pCAG-EGFP were from Addgene Inc (Cat#11,150).

#### Primary antibodies

Polyclonal rabbit anti-Myc was produced as described previously [17]. The following rabbit polyclonal antibodies were used: anti-Cux1 (Gene Tex, Cat# GTX56275, 1:300), anti-GFP (Medical & Biological Laboratories, Cat# 598, 1:1000), anti-Iba1 (FUJIFILM, Cat# 019–19741, 1:1000), anti-NeuN (Abcam, Cat# ab177487, 1:3000), and anti-Kv7.2 (KCNQ2; Synaptic Systems, Cat# 368 103, 1:1000). Polyclonal guinea pig anti-Ankyrin G was from Synaptic Systems (Cat# 368 005, 1:500). Mouse monoclonal anti-GFAP (Sigma, Cat# G3893, 1:300), and anti-β-actin (Cell Signaling, Cat# 3700, 1:5000), as well as rat monoclonal anti-Ctip2 antibody (Abcam, Cat# ab18465, 1:500), were also used. Alexa Fluor 488-, 568-, and 647-labeled IgG (Abcam, Cat# ab150077, ab175471, and ab150075, respectively) were used as secondary antibodies at a 1:1000 dilution. 4’, 6-diamidino-2-phenylindole (DAPI) (Sigma-Aldrich, Cat# D9542, 0.2 μg/ml) was used to stain DNA.

#### Structural analysis

To determine the protein structure of the human Kv7.2, we consulted with the AlphaFold Protein Structure Database (https://alphafold.ebi.ac.uk/), and performed a structural analysis using PyMol software (http://www.pymol.org/pymol/).

#### Generation of KI mice, ***Kcnq2***^R213W/+^ and ***Kcnq2***^R213Q/+^, carrying the c.637C > T, p.R213W and c.638G > A, p.R213Q variants, respectively

The KI mouse models, *Kcnq2*^R213W/+^ and *Kcnq2*^R213Q/+^, with Slc:ICR background were generated using the improved-genome editing via oviductal nucleic acids delivery (i-GONAD) technique [18]. For *Kcnq2*^R213W/+^ and *Kcnq2*^R213Q/+^, crRNA 5′- GGATGATCCGTATGGACCGG-3′ was chosen to target exon 4 (Supplementary Fig. 1A). ssODN (5′-TTGCGGTTCTTGCAAATCTTGCGGATGATCCGTATGGACTGGAGGGGTGGCACCTGGAAGCTCTTGG -3′ for R213W and 5′-TTGCGGTTCTTGCAAATCTTGCGGATGATCCGTATGGACCAGAGGGGTGGCACCTGGAAGCTCTTGG -3′ for R213Q, respectively), Cas9 (cat# 1,081,060), and tracrRNA (cat# 1,072,534) were obtained from Integrated DNA Technologies Inc. For the i-GONAD procedure, Cas9, crRNAs, and tracrRNA were dissolved in Opti-MEM and injected into the oviducts of Slc:ICR pregnant mice 0.5 day post conception. The electroporation parameters were as follows: poring pulse (voltage, 50 V; pulse length, 5 ms; pulse interval, 50 ms; number of pulses, 3; decay rate, 10%; polarity switch, +), and transfer pulse (voltage, 10 V; pulse length, 50 ms; pulse interval, 10 ms; number of pulses, 3; decay rate, 40%; polarity switch, ±). Genotyping for R213W was performed by digesting PCR products with PstI for R213W, and PvuII for R213Q, using genomic DNA extracted from the mice (R213W: forward primer, 5′-GGGCAATGTCTTTGCCACATCTGC-3′; reverse primer, 5′-GAGCTTCCAGGTGCCACCCCTGC-3′, R213Q: forward primer, 5′-GGGCAATGTCTTTGCCACATCTGC-3′; reverse primer, 5′-CCAAGAGCTTCCAGGTGCCACCCAGC-3′).

*Kcnq2*^R213W/+^ and *Kcnq2*^R213Q/+^ mice were independently generated and each backcrossed for at least five generations to C57BL/6 J. Thus, the lines are not strictly congenic, and their genetic backgrounds are not completely identical. For all analyses in this study, wild-type littermates from both lines were pooled and used as common controls.

#### Immunohistochemistry

Mice were deeply anesthetized with isoflurane inhalation, followed by perfusion with phosphate-buffered saline (PBS) and subsequently with 4% paraformaldehyde (PFA) in PBS.

After perfusion, the embryonic and adult brains were dissected and fixed in 4% PFA for at least.

16 h. Brains were then coronally sectioned at either 100 μm or 12 μm thickness. For immunostaining, embryonic brain sections were mounted on MAS-coated slides and treated with HistoVT One (Nacalai Tesque Inc., Cat#06380–05) at 70 °C for 20 min. After washing with PBS containing 0.05% Tween-20 (PBST), sections were blocked with 1% bovine serum alubmin in PBST. Primary antibodies were applied overnight at 4 °C in PBST. The following day, secondary antibody incubation and nuclear staining with DAPI (0.2 μg/ml) were carried out in PBST for 1 h. Stained sections were mounted using anti-fading mounting medium (PERMAFLUOR, Thermo Scientific, Cat# TA-030-FM). Fluorescence images were acquired using an LSM880 confocal laser microscope (Carl Zeiss), and bright field images were captured using a BZ-9000 microscope (Keyence).

#### Golgi–Cox staining and spine analysis

Golgi-Cox staining was performed using the FD Rapid GolgiStain Kit (FD NeuroTechnologies, Cat# PK401A) following the manufacturer’s protocol with slight modifications. One month-old male mice were deeply anesthetized with isoflurane and decapitated. Their brains were rapidly removed and placed in FD Solution AB (1:1 mixture) for two weeks at room temperature in the dark. After this incubation, the brains were transferred to FD Solution C or a tissue-protectant solution (20% sucrose, 15% glycerol in distilled water) and kept at 4 °C in the dark for 72 h before being coronally sectioned at 100 μm thickness. Sections were mounted and stained according to the manufacturer’s instructions. After dehydration, slides were mounted with Permount (Fisher Scientific, Cat# SP15-100). Z-stack images (20–30 stacks with 1 μm intervals for dendrites, 0.1 μm intervals for spines) were captured using 20x (dendrites) and 100x (spines) objectives on a BZ-9000 microscope. The number and length of basal dendritic branches were analyzed using the NeuronJ plugin in Fiji. Spine density on dendrites located within 50–100 μm of the cell body was quantified using the Dendritic Spine Counter plugin, also in Fiji.

#### In utero electroporation

In utero electroporation was conducted following a previously described protocol [19]. In brief, pregnant mice were deeply anesthetized with a mixture of butorphanol (5 mg/kg), medetomidine (0.75 mg/kg), and midazolam (4 mg/kg) [20]. The specified plasmid was injected into the lateral ventricles of the embryos, followed by electroporation using the NEPA21 electroporator (NEPA Gene). Electroporation parameters consisted of five 50 ms pulses at 35 V with 450 ms intervals. This method ensured plasmid introduction into the somatosensory cortex, located within the parietal lobe. After electroporation, brains were collected at the indicated embryonic or postnatal stages, fixed, sectioned, and subjected to analysis. All procedures were performed during daylight hours. No animals were excluded or harmed during the course of the experiments.

#### Electrophysiological analyses

The analyses were performed as described previously [21]. Briefly, coronal cortical slices (300 μm-thickness) from postnatal day (P) 12—19 mice were prepared in ice-cold cutting Krebs solution using a microslicer (PRO7, Dosaka, Kyoto, Japan)_._ The slices were transferred to a holding chamber containing standard Krebs solution and incubated at room temperature. For recording, a slice was superfused with standard Krebs solution at a rate of 3–4 ml/min. To record synaptic currents, patch pipettes made from borosilicate glass capillaries were filled with CsCl-based internal solution. For I_M_ and current-clamp recordings, patch pipettes were filled with K-gluconate based internal solution. Whole-cell recordings were conducted on pyramidal neurons in the cortical layer II/III and granule cells in dentate gyrus of the hippocampus using a patch-clamp amplifier (Axopatch 200B, Molecular Devices, Foster City, CA) and pCLAMP8 software (Molecular Devices). Miniature excitatory postsynaptic currents were recorded at a holding potential of -60 mV in the presence of bicuculline (10 μM, abcam, Cat# ab120110), strychnine (0.5 μM, Tokyo Chemical Industry, Cat# S0257) and tetrodotoxin (TTX) (0.5 μM, Wako, Cat# 206–11071). Miniature inhibitory postsynaptic currents were recorded at a holding potential of -60 mV in the presence of 6,7-Dinitroquinoxaline-2,3-dione (DNQX) (5 μM, Cayman Chemical Company, Cat# 14,583), D-AP5 (25 μM, Cayman Chemical Company, Cat# 14,539), strychnine (0.5 μM) and TTX (0.5 μM). To record the I_M_, the currents were held at -20 mV and hyperpolarizing steps during 3 s were applied from -20 to -60 mV in 10 mv-increments.

#### Gene expression analysis

Primary and secondary somatosensory areas from adult mice (P60) were dissected, and RNA was extracted using Sepasol®-RNA I Super (Nacalai Tesque Inc., Cat# G09379-97). The sample size was three males and three females per genotype (R213Q, R213W, and wild-type [WT]). mRNA libraries for sequencing were prepared using the MGIEasy RNA Directional Library Prep Set kit (MGI, Cat# 1,000,006,385), and sequencing was performed with the DNBSEQ-T7RS High-throughput Sequencing Kit (FCL PE150) V3.0 (MGI, Cat# 940–000266-00). RNA-seq reads were aligned to the mouse genome using the STAR aligner (v2.7.11), and gene-level read counts were obtained. Genes with low counts were filtered using the filterByExpr function. Normalization was performed using the trimmed mean of M-values (TMM), and differential expression analysis was conducted using a quasi-likelihood negative binomial generalized log-linear model (glmQLFit), with a model controlling for the animal sex. Genes with a false discovery rate (FDR) < 0.05 were considered significantly differentially expressed. Gene ontology (GO) enrichment analysis for differentially expressed genes (DEGs), including cellular components and biological process terms, was performed using ShinyGO (v0.82).

### Statistical analyses

For all imaging experiments, cell counting and morphological analyses were performed by an investigator blinded to the experimental conditions. Statistical analysis was carried out using GraphPad Prism 10 software (GraphPad Software Inc.). Data are presented as mean ± SD. For comparisons between two groups, Welch’s t-test was employed. For comparisons involving more than three groups, one-way analysis of variance (ANOVA) was performed, followed by a Tukey–Kramer least significant difference (LSD) test for post hoc analysis of multiple comparisons. A p-value of < 0.05 was considered statistically significant. Normality of data distribution was not assessed in this study. Box and whisker plots display the median (horizontal bars), the interquartile range (25th to 75th percentiles as box edges), and whiskers extending to the smallest and largest non-outlier values. The mean is indicated by the cross within the boxes.

## Results

### Generation of ***Kcnq2***^R213W/+^ and ***Kcnq2***^R213Q/+^ mice

Because the overall amino acid sequences of human and mouse Kv7.2 (NP_742105.1 and NP_034741.2, respectively) share 94.7% identity, and the region surrounding exon 4, where R213 is located is completely conserved (100% identity; Supplementary Fig. 1B), this high conservation provides a strong rationale for analyzing KI mice harboring mutations at this residue. Structural modeling further revealed that residue R213 forms hydrogen bonds with E86 and E140, and that substitution of R213 with W or Q disrupts these interactions (Supplementary Fig. 1C). Disruption of hydrogen bonding in membrane proteins is known to influence protein folding, assembly, and function [22–24]. Thus, the microstructural alterations caused by these substitutions may underlie functional changes in Kv7.2.

**Fig. 1:**
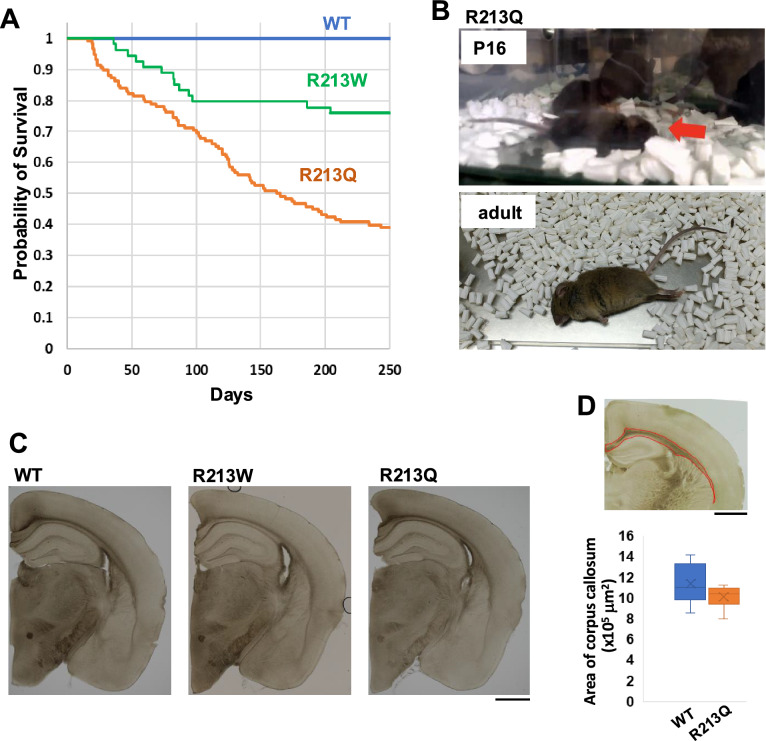
Characterization of *Kcnq2*^R213W/+^ and *Kcnq2*^R213Q/+^ mice. **A** Reduced survival in *Kcnq2*^R213Q/+^ mice. Kaplan–Meier survival curves of 131 WT, 74 *Kcnq2*^R213W/+^ and 118 *Kcnq2*^R213Q/+^ mice from P0 to P250. **B** Seizures in *Kcnq2*^R213Q/+^ mice. Representative images of seizure episodes at P16 (*upper* panel) and in adulthood (*lower* panel). The red arrow indicates a seizure captured at P16. See Supplementary video 1 for additional seizure events. **C** Normal brain morphology in the model mice. Bright field images of representative coronal sections from WT, *Kcnq2*^R213W/+^, and *Kcnq2*^R213Q/+^ mice at 7 months of age. **D** Quantification of corpus callosum size in WT and *Kcnq2*^R213Q/+^ mice at P21. The area enclosed by the red line was measured. *n* = 10 and 6 for WT and *Kcnq2*^R213Q/+^, respectively. Scale bars, 1 mm

To investigate the impact of the p.R213W and p.R213Q variants of *KCNQ2* on brain function, we generated KI mouse models, *Kcnq2*^R213W/+^ and *Kcnq2*^R213Q^ (Supplementary Fig. 1A and D). Both lines were born at Mendelian ratios (data not shown). Exploratory behavior in the home cage and circadian activity patterns were comparable to those of WT littermates. Kaplan–Meier survival curves revealed a significant reduction in lifespan for *Kcnq2*^R213Q/+^ mice compared with both WT and *Kcnq2*^R213W/+^ mice. *Kcnq2*^R213Q/+^ mice displayed progressive mortality, with 60% dying before 250 days of age (Fig. 1A). While WT mice exhibited high survival rates, approximately 20% of *Kcnq2*^R213W/+^ mice died between P40 and P100, with no substantial increase in mortality thereafter. Notably, deceased *Kcnq2*^R213Q/+^ and *Kcnq2*^R213W/+^ mice commonly exhibited a rigid posture with extended limbs and a severely curved spine—features highly indicative of epileptic seizures.

### Both ***Kcnq2***^R213W/+^ and ***Kcnq2***^R213Q/+^ mice exhibited tonic–clonic seizures

Seizures were observed in both *Kcnq2*^R213W/+^ and *Kcnq2*^R213Q/+^ mice. In *Kcnq2*^R213Q/+^ mice, the earliest seizure was visually observed at P8, and video-documented seizures were recorded from P16 (Fig. 1B; Supplementary video 1), indicating that the p.R213Q variant may disrupt neuronal excitability, leading to epileptic phenotypes in vivo. Seizure events were most frequently triggered by handling during home cage changes and were classified as generalized tonic–clonic seizures, consisting of distinct tonic and clonic phases (Fig. 1B; Supplementary video 2). Notably, death during seizures was frequently observed in *Kcnq2*^R213Q/+^ mice (Supplementary video 3). Although *Kcnq2*^R213W/+^ mice also exhibited generalized tonic–clonic seizures resembling those observed in *Kcnq2*^R213Q/+^, the seizure frequency was markedly lower. While some individuals in *Kcnq2*^R213Q/+^ mouse group exhibited recurrent seizures, *Kcnq2*^R213W/+^ mice were observed to have a single seizure at the time of death. Moreover, no seizures were observed during early developmental stages, and seizure incidence decreased further after P100 in *Kcnq2*^R213W/+^ mice (Fig. 1A; Supplementary video 4). Taken together, these findings indicate that the p.R213Q variant leads to a severe epilepsy phenotype characterized by high seizure frequency and reduced survival, whereas the p.R213W variant results in a milder phenotype with only a modest effect on lifespan.

### Effects of the p.R213W and p.R213Q variants on the Kv7.2 expression and gross brain morphology

To determine whether these epileptogenic *KCNQ2* variants affect protein expression levels, we performed western blot analysis using cortical lysates. Endogenous Kv7.2 expression is developmentally regulated in the mouse brain, with relatively low levels at E17 and P0, followed by a progressive increase from P3 to P60 (Supplementary Fig. 2A). To assess whether the p.R213W and p.R213Q variants affect protein stability, we expressed Myc-tagged WT and variant proteins in COS7 cells and found both variants to be as stable as WT (Supplementary Fig. 2B). We then compared endogenous Kv7.2 levels in the cortex of WT, *Kcnq2*^R213W/+^, and *Kcnq2*^R213Q/+^ mice. Western blotting revealed no significant differences in Kv7.2 abundance among the three genotypes, indicating that the severe epileptic phenotype observed in *Kcnq2*^R213Q/+^ mice appears not to be due to altered protein expression levels (Supplementary Fig. 2C).

Subsequently, we performed histological analysis to assess whether the p.R213W and p.R213Q variants lead to anatomical abnormalities. Representative coronal brain sections from WT, *Kcnq2*^R213W/+^, and *Kcnq2*^R213Q/+^ mice at 7 months of age revealed no apparent differences in overall brain structure, including cortical lamination and hippocampal organization (Fig. 1C; Supplementary Fig. 3A). The area of the corpus callosum was comparable between WT and *Kcnq2*^R213Q/+^ mice (Fig. 1D), suggesting that axon extension into the contralateral cortex is minimally affected by the mutation.

Taken together, despite the increased seizure susceptibility, severe epileptic phenotype, and reduced survival observed in *Kcnq2*^R213Q/+^ mice, no significant anatomical abnormalities were detected in the brain.

### Analyses of inflammatory changes in the hippocampus and cerebral cortex

Since no overt structural abnormalities were detected in cortical or hippocampal architecture, we next assessed potential neuroinflammatory changes associated with epilepsy. To this end, we evaluated glial responses to seizure activity by examining astrocytic and microglial activation using immunostaining for GFAP and Iba1, respectively. Brain sections from *Kcnq2*^R213Q/+^ mice aged 6–7 months were used, as four seizure episodes had been confirmed at this age. Notably, although the first seizure sometimes occurred earlier, subsequent seizures were infrequent, and many mice died after the second or third episode.

**Fig. 2:**
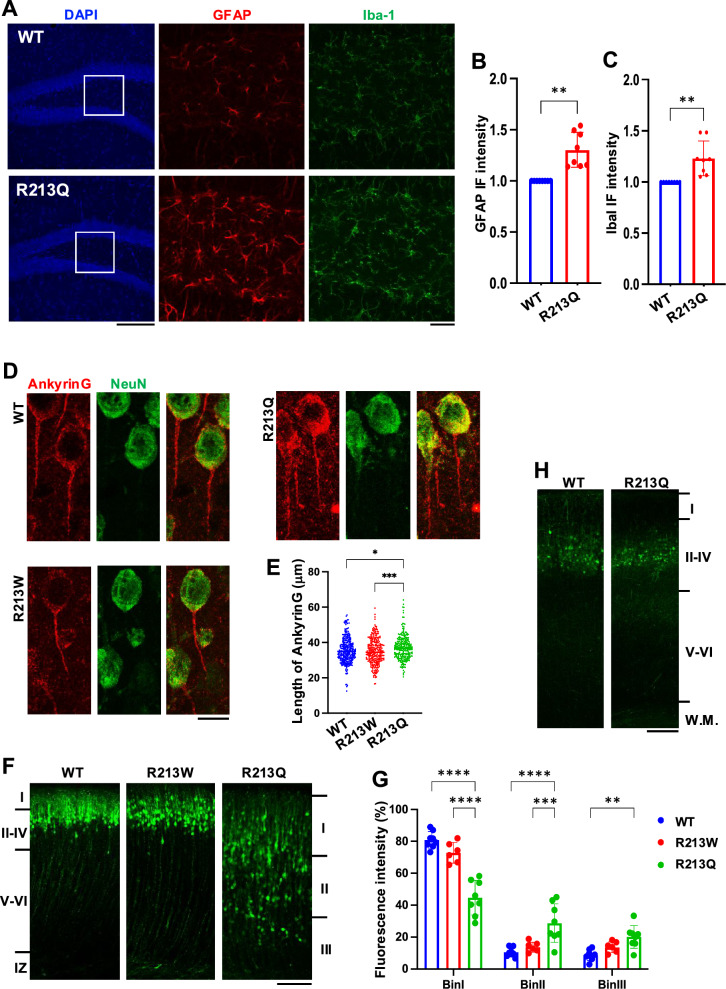
Immunohistochemical and morphological analysis of *Kcnq2*^R213W/+^ and *Kcnq2*^R213Q/+^ mice. **A** Increased gliosis in the dentate gyrus of *Kcnq2*^R213Q/+^ mice. Representative immunofluorescence images of the dentate gyrus from WT and *Kcnq2*^R213Q/+^ mice at 6–7 months of age. Sections were stained with DAPI (blue), GFAP (red), and Iba1 (green). Boxes in the DAPI panels indicate the areas magnified in the adjacent GFAP and Iba1 panels. Scale bars, 200 μm (left) and 50 μm (right). **B**, **C** Quantification of gliosis shown in (A). Bar graphs represent the immunofluorescence (IF) intensity of GFAP (B) and Iba1 **C** in the dentate gyrus of WT and *Kcnq2*^R213Q/+^ mice. Sample size: i = 8 per group. **p < 0.01. **D** AnkyrinG localization at the AIS of cortical neurons. Representative immunofluorescence images showing AnkyrinG (red) and NeuN (green) in the cortex of WT, *Kcnq2*^R213W/+^, and *Kcnq2*^R213Q/+^ mice at P30. Merged images are also shown. Scale bar, 10 μm. **E** Quantification of AIS length in **D**. Scatter plot showing AnkyrinG-positive AIS length in cortical neurons from each genotype. Sample size: *n* = 3 animals per genotype; 319, 295, and 261 cells for WT, *Kcnq2*^R213W/+^, and *Kcnq2*^R213Q/+^, respectively. *p < 0.05; ***p < 0.001. **F** Cortical neuron migration. Neurons were labeled by in utero electroporation at E14, and brains were analyzed at P0. Representative images of GFP-labeled neurons in the cortex of WT, *Kcnq2*^R213W/+^, and *Kcnq2*^R213Q/+^ mice. Cortical layers are indicated on the left. IZ, intermediate zone. Scale bar, 100 μm. **G** Quantification of (F). Graphs show the fluorescence intensity distribution of GFP-positive neurons across cortical bins (I–III). Sample size: *n* = 8, 6 and 8 for WT, *Kcnq2*^R213W/+^ and *Kcnq2*^R213Q/+^ mice, respectively. ****p < 0.0001; ***p < 0.001; **p < 0.01. (H) Representative images of cortical neuron migration of WT and *Kcnq2*^R213Q/+^ mice at P7. W.M., while matter. Scale bar, 100 μm

The results revealed that GFAP-positive astrocytes were markedly increased in the dentate gyrus of *Kcnq2*^R213Q/+^ mice (Fig. 2A and B), indicating astrocytic activation in response to chronic seizures. Similarly, expression of Iba1, a marker of activated microglia, was elevated in the same region (Fig. 2A and C), suggesting an inflammatory response associated with persistent seizure burden. In contrast, *Kcnq2*^R213W/+^ mice exhibited no elevation in GFAP or Iba1 expression (Supplementary Fig. 3B). These findings suggest that seizure activity in *Kcnq2*^R213Q/+^ mice—defined in this study as seizures most frequently triggered by routine handling procedures such as cage changes or feeding— induces astrocytic and microglial activation, contributing to neuroinflammation in the hippocampus. Notably, these neuroinflammatory changes in *Kcnq2*^R213Q/+^ mice were largely restricted to the hippocampus, as similar increases in GFAP expression were not observed in the cerebral cortex (Supplementary Fig. 3C). This region-specific gliosis suggests that the dentate gyrus is particularly vulnerable to seizure-induced neuroinflammation in *Kcnq2*^R213Q/+^ mice. Together, these findings indicate that the chronic seizures observed in *Kcnq2*^R213Q/+^ mice are associated with localized neuroinflammatory responses, including astrocyte and microglial activation, which may contribute to disease progression and neuronal dysfunction.

### Morphological analysis of the axon initial segment (AIS) in the mouse models

Given that Kv7.2 is highly enriched in the AIS of neurons, we examined its morphology by immunostaining for AnkyrinG, a structural marker of the AIS, in cortical neurons at P30. This analysis revealed that AnkyrinG-positive AIS structures were significantly elongated in *Kcnq2*^R213Q/+^ mice compared to WT and *Kcnq2*^R213W/+^ mice (Fig. 2D and E). In contrast, AIS morphology in *Kcnq2*^R213W/+^ mice appeared similar to that of WT, consistent with the relatively milder phenotype observed in these mice (Fig. 2D and E).

Given the observed structural alterations in the AIS, we hypothesized that cortical neurons in *Kcnq2*^R213Q/+^ mice might also exhibit morphological abnormalities at the cellular level, potentially affecting the formation and maintenance of synaptic networks. To test this, we analyzed the morphology of dendrites and synapses in Golgi-Cox-stained pyramidal neurons located in layer II/III of the somatosensory cortex at P60. Quantitative analysis revealed that both the number of branches and the total length of basal dendrites in *Kcnq2*^R213Q/+^ mice were comparable to those in control littermates (Supplementary Fig. 4A–C). Furthermore, spine densities in cortical pyramidal neurons, as well as in hippocampal neurons within the CA2 and CA3 regions, did not differ significantly between *Kcnq2*^R213Q/+^ mice and controls (Supplementary Fig. 4D–G).

### Effects of the p.R213W and p.R213Q variants on cortical neuron migration during corticogenesis

To assess whether the p.R213W and p.R213Q variants affect cortical neuron migration, we performed in utero electroporation at E14 and analyzed their positioning at P0. Fluorescence imaging revealed a significant migration delay in *Kcnq2*^R213Q/+^ mice compared to WT and *Kcnq2*^R213W/+^ mice (Fig. 2F). While the majority of GFP-labeled neurons in WT and *Kcnq2*^R213W/+^ mice had reached the upper cortical layers (layers II/III), many neurons in Kcnq2^R213Q/+^ mice remained in the deeper layers, indicating migration impairment (Fig. 2F and G). Notably, by P7, most GFP-labeled neurons in Kcnq2^R213Q/+^ mice had reached their appropriate laminar positions (Fig. 2H), suggesting that the migration defect is transient rather than permanent. Consistent with this, the laminar distribution of Cux1- and Ctip2-expressing neurons at P40 did not differ significantly among WT, *Kcnq2*^R213W/+^, and *Kcnq2*^R213Q/+^ mice (Supplementary Fig. 3A), indicating that overall cortical layer organization is ultimately preserved.

### Dysfunction of Kv7.2 channels and increased neuronal excitability in dentate gyrus granule cells of the mouse models

**Fig. 3:**
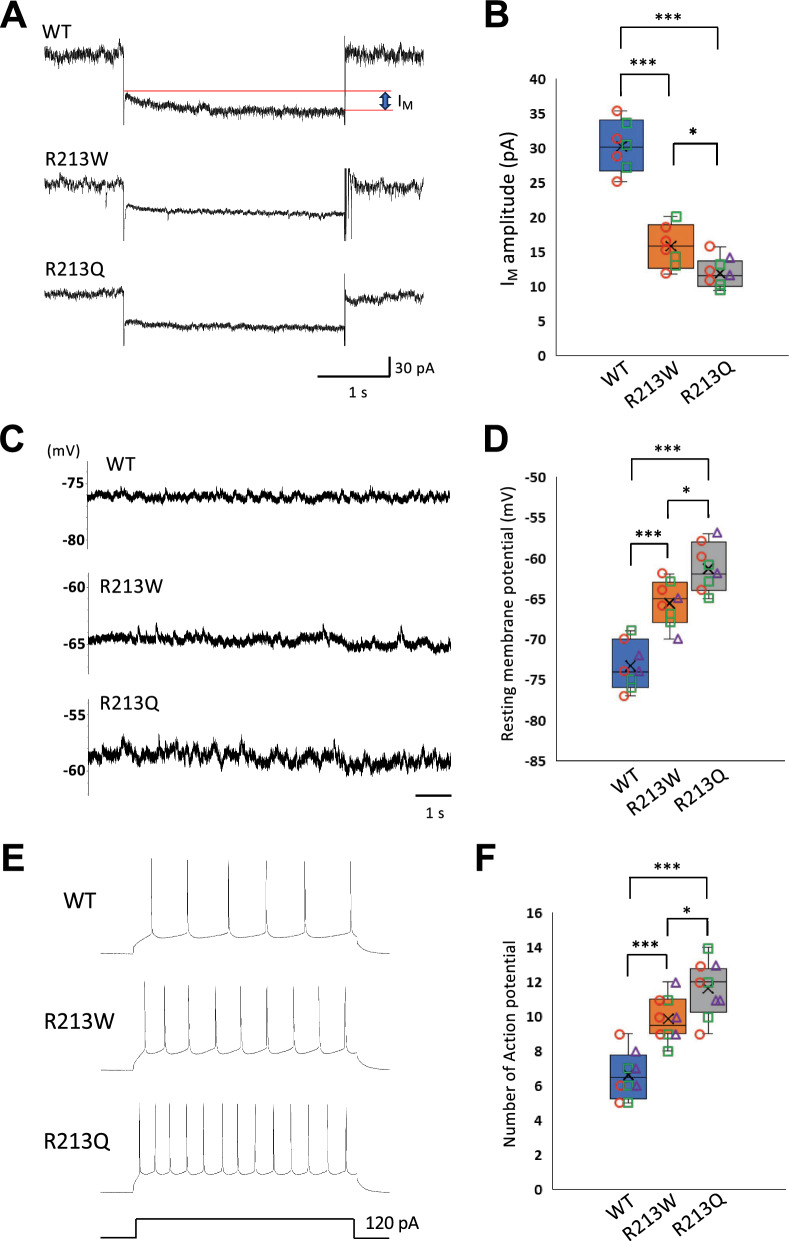
Impaired Kv7.2 channel function and increased neuronal excitability in granule cells of the hippocampal dentate gyrus from *Kcnq2*^R213W/+^ and *Kcnq2*^R213Q/+^ mice. **A** Representative traces of I_M_ recorded from granule cells in WT, *Kcnq2*^R213W/+^, and *Kcnq2*^R213Q/+^ mice. The voltage-clamp protocol was described previously, and the current traces show hyperpolarizing steps to  −40 mV from a holding potential of  −20 mV. **B** Quantification of **A**. Recordings were obtained from 7 WT neurons and 7 *Kcnq2*^R213W/+^ neurons (3 slices from 2 mice per genotype), and 8 *Kcnq2*^R213Q/+^ neurons (4 slices from 3 mice). **C** Representative recordings of RMP in granule cells from each genotype. **D** Quantification of **C**. Eight neurons were analyzed per genotype (4 slices from 3 mice per genotype). **E** Representative traces of action potentials evoked by a 120 pA current injection for 800 ms in granule cells from WT, *Kcnq2*^R213W/+^, and *Kcnq2*^R213Q/+^ mice. **F** Quantification of **E**. Recordings were obtained from 9 neurons per genotype (4 slices from 3 mice). The number of mice represents the biological replicates, whereas the number of slices and neurons represents technical replicates. Box plots show the median (horizontal line), interquartile range (box), and full range (whiskers). In the dot plots, different point shapes indicate the individual mice: red circles for mouse #1, green squares for mouse #2, and purple triangles for mouse #3. See “Materials and methods” for details. *P < 0.05; ***P < 0.001 (Tukey–Kramer LSD test)

To evaluate the functional impact of the p.R213W and p.R213Q variants on neuronal excitability, we performed whole-cell patch-clamp recordings from granule cells in the dentate gyrus of hippocampal slices obtained from WT, *Kcnq2*^R213W/+^, and *Kcnq2*^R213Q/+^ mice at P12-19. We first examined the I_M_, a subthreshold potassium current mediated by Kv7.2 channels, which plays a key role in stabilizing membrane potential and limiting neuronal excitability. Representative current traces revealed voltage-dependent activation of I_M_ in WT as well as in both mutant neurons (Fig. 3A). However, quantitative analysis showed a significant reduction in I_M_ amplitudes in both *Kcnq2*^R213W/+^ and *Kcnq2*^R213Q/+^ neurons compared to WT, with the reduction being more pronounced in the latter (Fig. 3A and B). We next measured the resting membrane potential (RMP). Both *Kcnq2*^R213W/+^ and *Kcnq2*^R213Q/+^ granule cells exhibited significantly depolarized RMPs relative to WT neurons, with Kcnq2^R213Q/+^ neurons showing a greater degree of depolarization (Fig. 3C and D). Together, these results indicate that both the p.R213W and p.R213Q variants impair Kv7.2 channel function, with the p.R213Q variant exerting a more severe effect, leading to elevated neuronal excitability. We next examined the excitability of granule cells by analyzing their firing rates. Action potentials were evoked by injecting a 120 pA current pulse for 800 ms. Both *Kcnq2*^R213W/+^ and *Kcnq2*^R213Q/+^ neurons exhibited significantly higher firing rates compared to WT neurons, with the increase being more pronounced in *Kcnq2*^R213Q^ neurons (Fig. 3E and F).

To determine whether synaptic transmission was altered, we recorded miniature excitatory postsynaptic currents (mEPSCs) and miniature inhibitory postsynaptic currents (mIPSCs) using voltage-clamp recordings. The frequency and amplitude of mEPSCs in both *Kcnq2*^R213W/+^ and *Kcnq2*^R213Q/+^ neurons were comparable to those in WT (Supplementary Fig. 5A and B). Similarly, no significant differences in the frequency or amplitude of mIPSCs were observed between WT and either mutant (Supplementary Fig. 5C and D). These findings indicate that the p.R213W and p.R213Q variants do not appreciably affect excitatory or inhibitory synaptic transmission, although previous work reported that Kv7.2 play a regulatory role in neurotransmitter release from rat hippocampal nerve ending [25].

In summary, both *Kcnq*2 variants result in marked neuronal hyperexcitability, likely driven by impaired I_M_ and depolarized RMP. The p.R213Q variant, however, causes a more severe dysfunction, which may underlie the epilepsy phenotypes observed in this model.

### Kv7.2 channel dysfunction and increased firing rate in cortical pyramidal neurons of ***Kcnq***2^R213Q/+^ mice

**Fig. 4:**
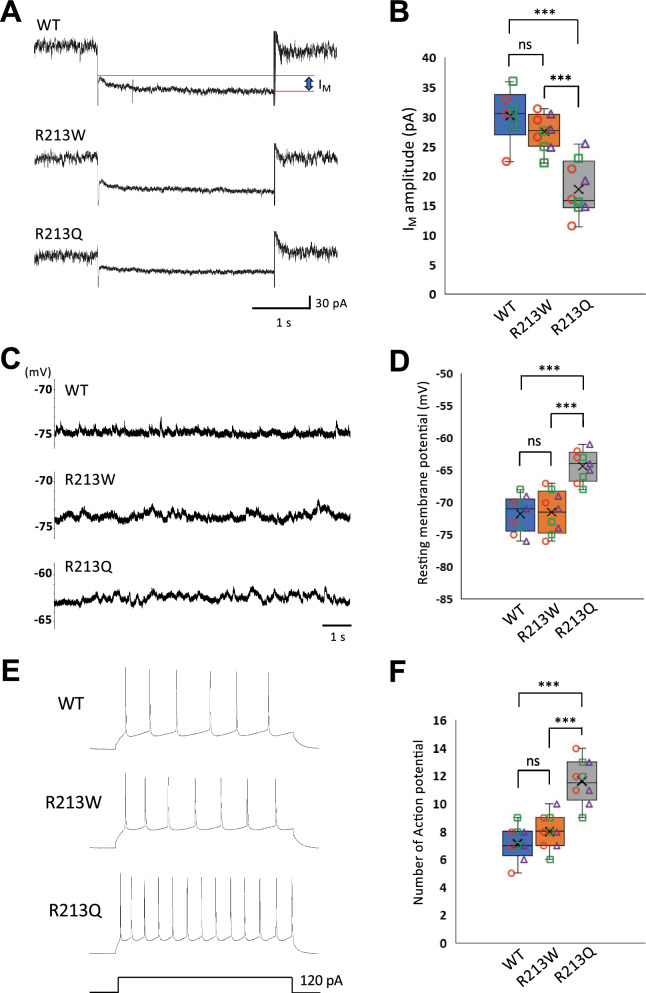
Dysfunction of Kv7.2 channels and increased firing rate in cortical neurons from *Kcnq2*^R213Q/+^ mice. **A** Representative traces of I_M_ recorded from layer II/III pyramidal neurons in WT, *Kcnq2*^R213W/+^, and *Kcnq2*^R213Q/+^ mice. The voltage-clamp protocol was described previously, and the current traces show hyperpolarizing steps to  −40 mV from a holding potential of -20 mV. **B** Quantification of **A**. Recordings were obtained from 7 WT neurons (3 slices from 2 mice) and 9 neurons each for *Kcnq2*^R213W/+^ and *Kcnq2*^R213Q/+^ genotypes (3 slices from 3 mice each). **C** Representative traces of RMPs recorded from cortical neurons in each genotype. **D** Quantification of **C**. Nine neurons were analyzed per genotype (3 slices from 3 mice each). **E** Representative traces of action potentials evoked by a 120 pA current injection (800 ms) in layer II/III pyramidal neurons from WT, *Kcnq2*^R213W/+^, and *Kcnq2*^R213Q/+^ mice. **F** Quantification of **E**. Recordings were obtained from 9 neurons per genotype (3 slices from 3 mice). The number of mice represents the biological replicates, whereas the number of slices and neurons represents technical replicates. Box plots show the median (horizontal line), interquartile range (box), and full range (whiskers). In the dot plots, different point shapes indicate the individual mice: red circles for mouse #1, green squares for mouse #2, and purple triangles for mouse #3. See “Materials and methods” for details. *P < 0.05; ***P < 0.001 (Tukey–Kramer LSD test)

To assess the impact of the p.R213W and p.R213Q variants on cortical excitability, we performed whole-cell patch-clamp recordings from layer II/III pyramidal neurons at P12-19. The amplitude of the I_M_ was significantly reduced in *Kcnq2*^R213Q/+^ neurons compared to WT, whereas I_M_ in *Kcnq2*^R213W/+^ neurons was comparable to WT (Fig. 4A and B). These results indicate that the p.R213Q variant markedly impairs Kv7.2 channel function in pyramidal neurons, while the p.R213W variant has little effect under the conditions tested. We next assessed RMP in the same neuronal population. Pyramidal neurons from *Kcnq2*^R213Q/+^ mice exhibited a significantly depolarized RMP compared to both WT and Kcnq2^R213W/+^ neurons, whereas *Kcnq2*^R213W/+^ neurons did not differ significantly from WT (Fig. 4C and D). This indicates that only the p.R213Q variant substantially alters RMP in cortical neurons. Collectively, these findings demonstrate that the p.R213Q variant causes notable deficits in Kv7.2 channel function and neuronal excitability in the cortex, whereas the p.R213W variant exerts minimal functional effects in this brain region.

To further investigate intrinsic excitability, we examined action potential firing properties by injecting depolarizing current pulses. *Kcnq*2^R213Q/+^ neurons showed a significantly higher firing rate than both WT and *Kcnq2*^R213W/+^ neurons, while *Kcnq2*^R213W/+^ neurons were comparable to WT (Fig. 4E and F). These results demonstrate that increased excitability is specific to the p.R213Q variant. These findings indicate that the p.R213Q variant enhances intrinsic neuronal excitability, likely due to impaired Kv7.2 channel function and depolarized RMP, whereas the p.R213W variant exerts minimal effects. Then, to assess whether synaptic transmission was altered, we recorded mEPSCs and mIPSCs using voltage-clamp recordings. Both the frequency and amplitude of mEPSCs and mIPSCs in neurons from *Kcnq2*^R213W/+^ and *Kcnq2*^R213Q/+^ mice were not significantly different from those in WT neurons (Supplementary Fig. 6A—D), indicating that excitatory and inhibitory synaptic inputs onto cortical neurons remain largely unaffected by either variant. Thus, neither the p.R213W nor p.R213Q variant alters baseline synaptic transmission in this region.

Collectively, these results demonstrate that the p.R213Q variant leads to marked neuronal hyperexcitability in the cortex, likely resulting from impaired I_M_ and RMP depolarization, without affecting synaptic transmission. In contrast, the p.R213W variant has little impact on either intrinsic excitability or synaptic function, consistent with its association with a milder, benign epilepsy phenotype.

### Gene expression analysis

**Fig. 5:**
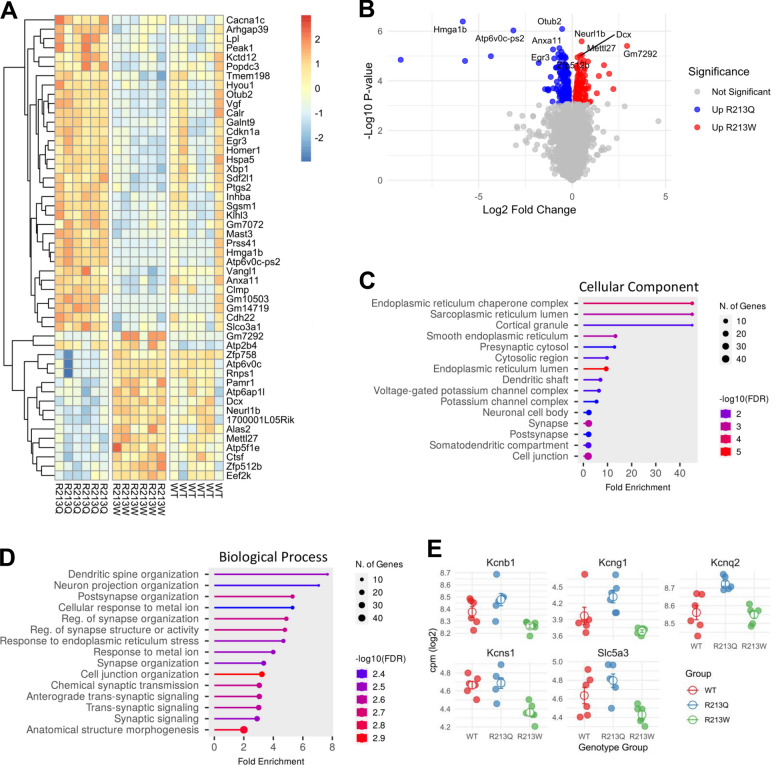
Differential gene expression analysis reveals transcriptomic differences between *Kcnq2*^*R213W/*+^ and *Kcnq2*^*R213Q/*+^ mice. **A** Heatmap of the top 50 most significant DEGs across WT, *Kcnq2*^*R213W/*+^, and *Kcnq2*^*R213Q/*+^ mice. Gene expression values are log2-transformed CPM (counts per million), scaled, and clustered by genes. **B** Volcano plot of gene expression differences between *Kcnq2*^R213W/+^ and *Kcnq2*^R213Q/+^ mice. DEGs (FDR < 0.05) are colored according to direction of change: genes upregulated in *Kcnq2*^R213W^ are shown in red, genes upregulated in *Kcnq2*^R213Q/+^ in blue, and non-significant genes in gray. Selected top 5 genes upregulated in each group (based on p-value) are labeled. Fold-change is shown on the x-axis (log₂ scale) and significance on the y-axis as -log₁₀ (p-value). (**C**, **D**) GO enrichment analysis for genes upregulated in *Kcnq2*^R213Q/+^ relative to *Kcnq2*^R213W/+^. Enrichment of top 15 cellular component terms **C**, and top 15 biological process terms **D**. **E** Expression of voltage-gated potassium channel subunits. Each point represents the log-transformed CPM values for an individual sample, colored by genotype. The empty point represents the mean and the error bars are ± standard error of the mean (SEM)

To investigate transcriptomic differences between the genotypes, we performed DEGs analysis comparing WT, *Kcnq2*^R213Q/+^, and *Kcnq2*^R213W/+^ mice, while accounting for sex as a covariate, using a quasi-likelihood generalized linear model across the three genotypes. This analysis identified 107 DEGs with a false discovery rate (FDR) < 0.05 (Supplementary Table S1). Hierarchical clustering of these DEGs revealed that the expression differences were primarily driven by the contrast between *Kcnq2*^R213Q/+^ and *Kcnq2*^R213W/+^ mice (Fig. 5A). Consistent with this, no genes met the significance threshold in pairwise comparisons between WT and either mutant when adjusted for multiple testing, suggesting a high degree of overlap at the global level. However, a direct comparison between *Kcnq2*^R213Q/+^ and *Kcnq2*^R213W/+^ revealed 267 DEGs (FDR < 0.05), including 159 genes upregulated in *Kcnq2*^R213Q/+^ and 108 genes upregulated in *Kcnq2*^R213W/+^ (Fig. 5B, Supplementary Table S2). We confirmed the upregulation of *Prss41* and *Mettl27* in *Kcnq2*^R213Q^ and *Kcnq2*^R213W/+^ mice, respectively, using quantitative RT-PCR (Supplementary Fig. 7A and B).

GO enrichment analysis identified significantly enriched terms (FDR < 0.05) exclusively among genes upregulated in *Kcnq2*^R213Q/+^ relative to *Kcnq2*^R213W/+^. These enriched terms were mainly associated with endoplasmic reticulum (ER) stress response (evidenced by upregulation of *Calr*, *Hspa5*, and *Xbp1*, etc.) and synaptic regulation pathways (Fig. 5C and D, Supplementary Table S3). In addition, there was enrichment for genes coding for subunits of voltage-gated potassium channels (i.e., *Kcns1*, *Kcnb1*, *Kcng1*, and *Kcnq2*). Notably, both *Kcnq2* and *Kcng1* were significantly upregulated in *Kcnq2*^R213Q/+^ mice compared to *Kcnq2*^R213W/+^ and WT (Fig. 5E). Furthermore, *Slc5a3*, which encodes the Na^+^/myo-inositol cotransporter known to associate with the Kv7.2/Kv7.3 channel and alter ion selectivity [26], was also upregulated (Fig. 5E). Quantitative RT-PCR further confirmed the upregulation and downregulation of genes involved in synaptic regulation (*Erg3* and *Homer1*), the ER stress response (*Sdf2l1*), and potassium channel function (*Slc5a3* and *Kcng1*) (Supplementary Fig. 7C- H).

## Discussion

We generated *Kcnq2* KI mice carrying the p.R213Q or p.R213W variant to examine their respective contributions to EIDEE and SeLFNIE. Although both *Kcnq2*^R213W/+^ and *Kcnq2*^R213Q/+^ mice showed normal gross brain morphology, cortical lamination, and hippocampal formation, gliosis was specifically observed in the dentate gyrus of *Kcnq2*^R213Q/+^ mice (Fig. 2A–C), suggesting that hippocampal neurons may be more vulnerable to the p.R213Q variant, whereas cortical neurons are intrinsically more resilient. We next focused on the AIS, a site of Kv7.2 enrichment essential for action potential initiation [27, 28]. In *Kcnq2*^R213Q/+^ mice, but not in *Kcnq2*^R213W/+^ mice, the AIS was abnormally elongated (Fig. 2D and E), which may account for their aberrant electrophysiological properties. The p.R213Q variant is likely to impair AIS-dependent excitability in cortical neurons, contributing to the pathogenesis of EIDEE. Alternatively, altered neuronal activity could secondarily induce AIS elongation, consistent with AIS plasticity [29].

Although the cortical layer structure of *Kcnq2*^R213W/+^ and *Kcnq2*^R213Q/+^ mice appeared grossly normal in adulthood, we found that functional defects in *Kcnq2*^R213Q/+^ mice led to delayed neuronal migration during corticogenesis, suggesting that Kv7.2 plays a crucial role in the establishment of cortical architecture in the embryonic stage. Given its predominant expression during early developmental stages [1], Kv7.2 is presumed to function as homotetramers at this time. Thus, a specific subunit composition (Kv7.2: Kv7.2-R213Q = 2:2) may dysregulate not only channel properties but also cytoskeletal dynamics, thereby impairing the migration of excitatory neurons during cortical development.

Consistent with these findings, microscopic cortical malformations have been reported in a patient with intractable neonatal seizures carrying the p.G215R variant, which is located in close proximity to R213 [16]. Histopathological examination of the patient’s autopsy samples revealed abnormal clustering of cortical neurons in layers V and VI of the frontal lobes, despite the preservation of macroscopic brain structures such as the neocortical gyral pattern [16]. In addition, the interface between the cortex and underlying white matter appeared diffusely blurred due to the presence of numerous heterotopic neurons, accompanied by marked reactive gliosis in the affected regions [16]. These histopathological features resemble the aberrant neuronal migration and glial activation observed in *Kcnq2*^R213Q/+^ mice, further supporting the face validity of this KI model for studying *KCNQ2*-related cortical malformations. Another EIDEE model, *Kcnq2*^T274M/+^ mice, exhibited phenotypes similar to those of *Kcnq2*^R213Q/+^, including increased early mortality and the absence of gross morphological abnormalities, although electrophysiological analyses were not performed [30]. Notably, cortical pyramidal neuron–specific *Kcnq2* cKO mice also showed cortical hyperexcitability and early death [31], while heterozygous loss of *Kcnq2* alters social, repetitive, and exploratory behaviors [32]. These findings underscore the indispensable function of Kv7.2, the activity of which is under strict regulation.

Electrophysiological analyses in heterologous systems, such as CHO cells and *Xenopus* oocytes, have shown that the p.R213Q variant induces more severe functional impairment than p.R213W, disrupting channel gating and increasing neuronal excitability, findings consistent with the clinical phenotype associated with p.R213Q [5, 6]. Computational modeling further revealed enhanced firing frequency of hippocampal neurons carrying p.R213Q [5], while *Xenopus* studies demonstrated a depolarizing shift in activation and reduced current amplitudes despite proper surface expression of Kv7.2-R213Q [6]. Although some discrepancies exist across assay systems, the p.R213Q variant is likely to substantially impair channel opening and exert dominant-negative effects on Kv7.2/Kv7.3 subunits, particularly at subthreshold voltages, underpinning the severe clinical phenotypes.

Our KI mouse models provide in vivo validation of the mechanisms by which the p.R213W and p.R213Q variants disrupt neuronal excitability. Both variants exhibited reduced I_M_ amplitude, depolarized RMP, and increased action potential firing in dentate granule cells, with more pronounced effects in *Kcnq2*^R213Q/+^ mice (Fig. 3). In contrast, cortical pyramidal neurons were affected only by p.R213Q (Fig. 4), which also exhibited abnormal AIS elongation (Fig. 2D). Given the little effects on the frequency and amplitude of spontaneous synaptic events (Supplementary Figs. 5 and 6), the enhanced excitability in *Kcnq2*^R213Q/+^ neurons may be attributable intrinsic membrane properties (impaired I_M_ and depolarized RMP).

These region- and variant-specific alterations not only align with previous in vitro findings but also extend them, demonstrating how p.R213Q uniquely disrupts neuronal function in vivo. Considering that SeLFNIE typically resolves within the first six months of life, cortical neuronal function may remain largely preserved in *Kcnq2*^R213W/+^ mice. In contrast, sustained hyperexcitability in cortical neurons—as observed with p.R213Q and another EIDEE-associated variant, p.I205V [33]—likely represents a key pathogenic mechanism in EIDEE. Furthermore, the hippocampus-specific gliosis in *Kcnq2*^R213Q/+^ mice supports the notion that regional vulnerability contributes to the phenotypic spectrum from SeLFNIE to EIDEE.

RNA-seq analysis identified differentially expressed genes that primarily reflected differences between *Kcnq2*^R213Q/+^ and *Kcnq2*^R213W/+^ mice (Fig. 5A). Despite the presence of dysregulated neuronal activity observed in *Kcnq2*^R213Q/+^ mice, GO analysis revealed that the most significantly upregulated pathways were related to the ER stress response and synaptic regulation (Fig. 5B). While EIDEE-associated *KCNQ2* variants may trigger cellular stress responses, the upregulation of synapse-related genes may represent compensatory mechanisms aimed at preserving synaptic integrity under pathological conditions. These transcriptomic findings provide novel insights into how pathogenic *KCNQ2* variants may influence neurodevelopment, not only by affecting neuronal excitability, but also by engaging stress-responsive and synaptic regulatory pathways. Notably, *Kcnq2* and *Kcng1* were significantly upregulated in *Kcnq2*^R213Q/+^ mice (Fig. 5C). It remains to be elucidated whether the p.R213Q variant alters the gating properties of Kv6.1-containing channels and reduces potassium conductance. On the other hand, since the Kv7.2 protein level was comparable to WT in *Kcnq2*^R213Q/+^ mice (Supplementary Fig. 2B and C), pathophysiological meaning of *Kcnq2* upregulation is unknown.

In conclusion, p.R213W and p.R213Q differentially perturb neuronal excitability in a region-dependent manner: both induce hyperexcitability in dentate granule cells, whereas p.R213Q alone causes cortical hyperexcitability accompanied by AIS elongation and ER stress signatures. These findings explain the SeLFNIE–EIDEE divergence and provide a platform for targeted interventions. *Kcnq2*^R213W/+^ and *Kcnq2*^R213Q/+^ mice recapitulate key aspects of human disease and serve as valuable models for dissecting *KCNQ2* pathophysiology. Continued investigation using these models may ultimately facilitate the development of precision therapies tailored to specific *KCNQ2* variant profiles.

## Supplementary Information

Additional file1 (PDF 156 KB). Supplementary Fig. 1 Strategy for generating Kcnq2R213W/+ and Kcnq2R213Q/+ mice using i-GONAD. (A) Targeted mutations were introduced into exon 4 of the Kcnq2 gene. Guide RNA sequence and corresponding proto-spacer adjacent motif (PAM) are shown. (B) Alignment of the amino acid sequences of human (NP_742105.1; upper row) and mouse (NP_034741.2; lower row) Kv7.2, illustrating the conservation of the region surrounding exon 4, where the KI mutations were introduced. Numbers indicate amino acid positions. (C) Structural models of wild-type human Kv7.2 (p.R213) and its variants p.R213W and p.R213Q. The structures, obtained from the AlphaFold Protein Structure Database (AF-O43526-F1-v4), were visualized using PyMOL software. Nitrogen atoms are shown in blue, oxygen atoms in red, and hydrogen bonds between amino acids are depicted as yellow dashed lines. (D) Direct sequencing results of the p.R213W (left) and p.R213Q (right). Corresponding amino acid sequences are shown for each.

Additional file2 (PDF 364 KB). Supplementary Fig. 2. Expression analysis of Kv7.2 and the p.R213W and p.R213Q variants. (A) Developmental expression profile of endogenous Kv7.2 in the mouse cerebral cortex. Western blot analysis (7.5 % gel) was performed using cortical lysates collected at various developmental stages (E17 to P60). GFAP and β-actin were used as an astrocyte marker and loading control, respectively. (B) Expression of Myc-tagged Kcnq2 constructs in COS7 cells. Cells were transfected with pCAG-Myc-Kcnq2 (WT), -Kcnq2-R213W, or -Kcnq2-R213Q (1 μg each). After 48h of incubation, cell lysates were collected and immunoblotted with anti-Myc. (C) Kv7.2 protein levels in Kcnq2R213W/+ and Kcnq2R213Q/+ mice at P34. Western blot analysis with anti-KCNQ2 (10% gel) of cortical lysates from WT, Kcnq2R213W/+, and Kcnq2R213Q/+ mice. β-actin was used to confirm equal protein loading.

Additional file3 (PPTX 1851 KB). Supplementary Fig. 3. Morphological analyses of the cerebral cortex and hippocampus. (A) Cortical layer organization at P40. Immunostaining was performed using Cux1 (upper-layer marker) and Ctip2 (deep-layer marker), showing normal laminar structure in Kcnq2R213W/+ and Kcnq2R213Q/+ mice. (B) Representative immunofluorescence images of the dentate gyrus from WT and Kcnq2R213W/+ mice at 6–7 months of age. Sections were stained as in Fig. 2A. Boxes in the DAPI panels indicate the areas magnified in the adjacent GFAP and Iba1 panels. Scale bars, 200 μm (left) and 50 μm (right). (C) Distribution of GFAP-positive astrocytes in the cerebral cortex of a 7-month-old Kcnq2R213Q/+ mouse that experienced four seizure episodes. Scale bar, 100 μm.

Additional file4 (PDF 206 KB). Supplementary Fig. 4. Morphological analyses of cortical and hippocampal neurons in Kcnq2R213Q/+ mice. (A) Dendritic morphology of cortical neurons. Representative images of Golgi-stained cortical neurons from WT and Kcnq2R213Q/+ mice (upper panel) at P30. Digitally reconstructed dendritic structures are shown in the lower panels. (B, C) Quantification of (A). Violin plots showing the number of dendritic branches (B) and the total basal dendritic length (C) in WT and Kcnq2R213Q/+ mice. n = 4 animals per genotype; 36 and 30 cells for WT and Kcnq2R213Q/+, respectively. No significant differences (ns) were observed. (D) Dendritic spine density in cortical neurons. Representative images of dendritic spines in cortical neurons from WT and Kcnq2R213Q/+ mice. (E) Quantification of (D). Violin plots showing dendritic spine densities (spines per 10 μm) in cortical neurons. n = 4 animals per genotype; 41 and 34 cells for WT and Kcnq2R213Q/+, respectively. No significant differences were detected. (F) Dendritic spine density in hippocampal CA2 (left) and CA3 (right) neurons. Representative images of dendritic spines in neurons from WT and Kcnq2R213Q/+ mice were shown. (G) Quantification of (F). Violin plots showing dendritic spine densities (spine number per 10 μm) in hippocampal CA2 neurons (n = 4 animals per genotype; 30 and 28 cells for WT and Kcnq2R213Q/+, respectively) and CA3 neurons (n = 4 animals per genotype; 27 and 30 cells for WT and Kcnq2R213Q/+, respectively). No significant differences were observed. Scale bar, 50μm (A) and 5μm (D and F).

Additional file5 (PDF 354 KB). Supplementary Fig. 5. Synaptic transmission in granule cells of the hippocampal dentate gyrus from Kcnq2R213W/+ and Kcnq2R213Q/+ mice. (A) Representative traces of mEPSCs recorded under the pharmacological conditions described in the “Materials and methods” section. (B) Quantification of mEPSC frequency and amplitude in (A). Recordings were obtained from 6 neurons per genotype (3 slices from 2 mice). (C) Representative traces of mIPSCs recorded under the conditions indicated in “Materials and methods” section. (D) Quantification of mIPSC frequency and amplitude in (C). Recordings were obtained from 6 neurons per genotype (2 slices from 2 mice). The number of mice represents the biological replicates, whereas the number of slices and neurons represents technical replicates. Box plots show the median (horizontal line), interquartile range (box), and full range (whiskers). In the dot plots, different point shapes indicate the individual mice: red circles for mouse #1, squares for mouse #2, and triangles for mouse #3. See “Materials and methods” for details. *P < 0.05; ***P < 0.001 (Tukey-Kramer LSD test).

Additional file6 (PDF 220 KB). Supplementary Fig. 6. Synaptic transmission in cortical neurons from Kcnq2R213W/+ and Kcnq2R213Q/+ mice. (A) Representative traces of mEPSCs recorded under the pharmacological conditions described in the “Materials and methods” section. (B) Quantification of mEPSC frequency and amplitude in (A). Recordings were obtained from 6 neurons per genotype (3 slices from 2 mice). (C) Representative traces of mIPSCs recorded under the pharmacological conditions described in the “Materials and methods” section. (D) Quantification of mIPSC frequency and amplitude in (C). Recordings were obtained from 6 neurons per genotype (3 slices from 2 mice). The number of mice represents the biological replicates, whereas the number of slices and neurons represents technical replicates. Box plots show the median (horizontal line), interquartile range (box), and full range (whiskers). In the dot plots, different point shapes indicate the individual mice: circles for mouse #1, squares for mouse #2, and triangles for mouse #3. See “Materials and methods” for details. *P < 0.05; ***P < 0.001 (Tukey-Kramer LSD test).

Additional file7 (PDF 218 KB). Supplementary Fig. 7. Quantitative RT-PCR analysis of the differentially expressed genes identified by RNAseq. Relative expression levels of Press41 (A), Mettl27 (B), Egr3 (C), Homer1 (D), Sdf2l1 (E), Hspa5 (F), Slc5a3 (G), and Kcng1 (H) were examined. cDNA was synthesized from total brain RNA using ReverTra Ace reagent (Toyobo, Cat#FSQ-301, Osaka, Japan). qPCR was performed with Thunderbird SYBR qPCR mix (Toyobo, Cat#QPS-201) on a CFX real-time PCR system (Bio-Rad Laboratories, GmbH, Germany) under the following conditions: 95°C for 1 min, followed by 40 cycles of 95°C for 15 s and 60°C for 45 s. Primer sequences were as follows: Press41 (forward: 5’-CTTGAAGAAGTCCCACCGCT-3’, reverse: 5’-GGACGGTCCACTTTTCTGGA-3’), Mettl27 (forward: 5’-GGAACCTCCCACGGTATCAC-3’, reverse: 5’-GTGGGGCTCGGTACTTCAAA-3’), Egr3 (forward: 5’-GATGGCTACAGAGAATGTGATGGA-3’, reverse: 5’- AGTCGAAAGCGAACTTTCCCA-3’), Homer1 (forward: 5’-AGTTTGGCCAATGGGCTGAT-3’, reverse: 5’- GCGACTTCTCCTTTGCAAGC-3’), Sdf2l1 (forward: 5’-CCTCTGTGTTCCTGTCGGTC-3’, reverse: 5’- CCATTGCCTTCCACGTGTTG-3’), Hspa5 (forward: 5’-CGGCTTCCGATAATCAGCCA-3’, reverse: 5’- TCAATCTGGGGAACTCCACG-3’), Slc5a3 (forward: 5’-TGCAGCGAGAATAGCGAAGT-3’, reverse: 5’- ATGACCCATGGAAGCCACTG-3’), Kcng1 (forward: 5’-CCTGGATGAGTTCCCACTGAC-3’, reverse: 5’- GGATGGTGCCAAAAGCTCCG-3’), and GAPDH (forward: 5’-TGATGGGTGTGAACCACGAGAA-3’, reverse: 5’-GGCATGGACTGTGGTCATGAG-3’). Relative expression levels (fold change), normalized to GAPDH and expressed relative to WT, were calculated using the ∆∆Ct method. Differences between R213Q and R213W were assessed by two-tailed unpaired t-test (Prism 10). Press41 (RQ>RW, ***p=0.0001), Mettl27 (RQ<RW, **p=0.0057), Egr3 (RQ>RW, *p=0.0318), Homer1 (RQ>RW, **p=0.0045), Sdf2l1 (RQ>RW, *p=0.0201), Hspa5 (ns, p=0.2126), Slc5a3 (RQ>RW, **p=0.0074), and Kcng1 (RQ>RW, **p=0.0037).

Additional file8 (AVI 9887 KB). Supplementary video 1 Seizures in Kcnq2R213Q/+ mice at P16.

Additional file9 (CSV 1857 KB)

Additional file10 (CSV 1109 KB)

Additional file11 (XLSX 53 KB)

Additional file12 (AVI 21583 KB). Supplementary video 2. Seizures in Kcnq2R213Q/+ mice in adult age.

Additional file13 (AVI 13749 KB). Supplementary video 3. Seizures in Kcnq2R213Q/+ mice in adult age.

Additional file14 (AVI 23039 KB). Supplementary video 4. Seizures in Kcnq2R213W/+ mice in adult age.

## Data Availability

The datasets used and/or analysed during the current study available from the corresponding author on reasonable request.
