# Supplementary figures and images for: Kcnq2 R213 knock-in mice reveal variant- and region-specific mechanisms underlying self-limited familial neonatal-infantile epilepsy and early infantile developmental and epileptic encephalopathy
Source: Acta Neuropathol Commun. 2026 Feb 25;14:76. doi: 10.1186/s40478-026-02264-4 (PMC13041443; doi:10.1186/s40478-026-02264-4)

**A** N47P031-Prss41

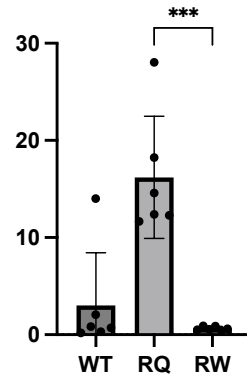

**B** N47P031-Mettl27

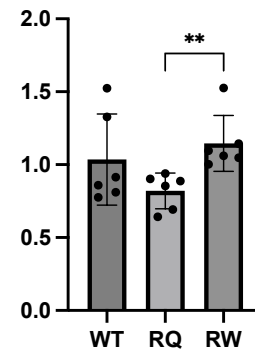

**C** Erg3

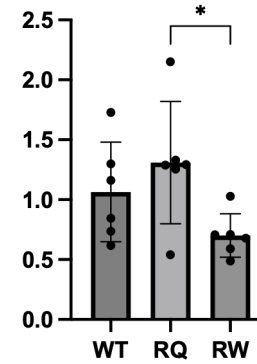

**D** Homer1

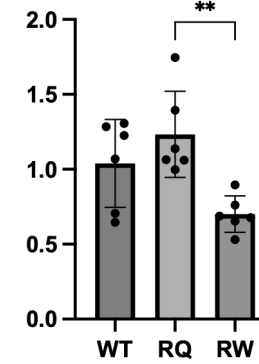

**E** Sdf2l1

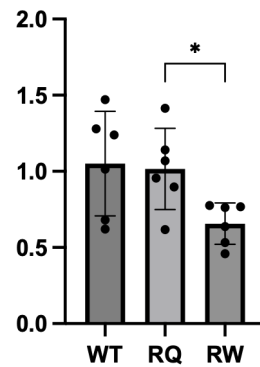

**F** Hspa5

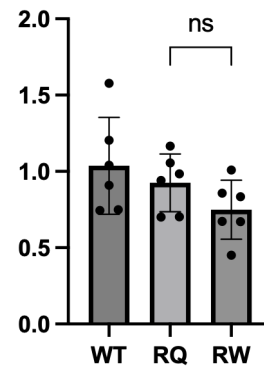

**G** Slc5a3

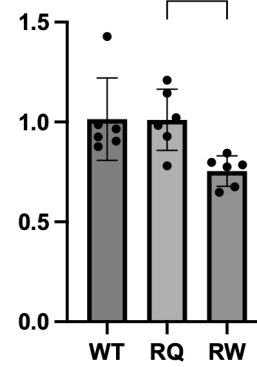

**H** Kcng1

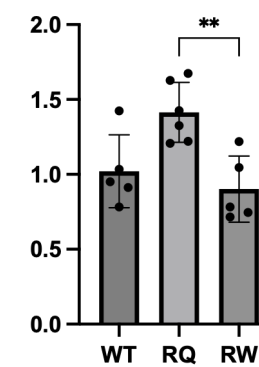

Supplement: Supplementary file 1 — Additional file1 (PDF 156 KB). Supplementary Fig. 1 Strategy for generating Kcnq2R213W/+ and Kcnq2R213Q/+ mice using i-GONAD. (A) Targeted mutations were introduced into exon 4 of the Kcnq2 gene. Guide RNA sequence and corresponding proto-spacer adjacent motif (PAM) are shown. (B) Alignment of the amino acid sequences of human (NP_742105.1; upper row) and mouse (NP_034741.2; lower row) Kv7.2, illustrating the conservation of the region surrounding exon 4, where the KI mutations were introduced. Numbers indicate amino acid positions. (C) Structural models of wild-type human Kv7.2 (p.R213) and its variants p.R213W and p.R213Q. The structures, obtained from the AlphaFold Protein Structure Database (AF-O43526-F1-v4), were visualized using PyMOL software. Nitrogen atoms are shown in blue, oxygen atoms in red, and hydrogen bonds between amino acids are depicted as yellow dashed lines. (D) Direct sequencing results of the p.R213W (left) and p.R213Q (right). Corresponding amino acid sequences are shown for each. [file 40478_2026_2264_MOESM1_ESM.pdf]

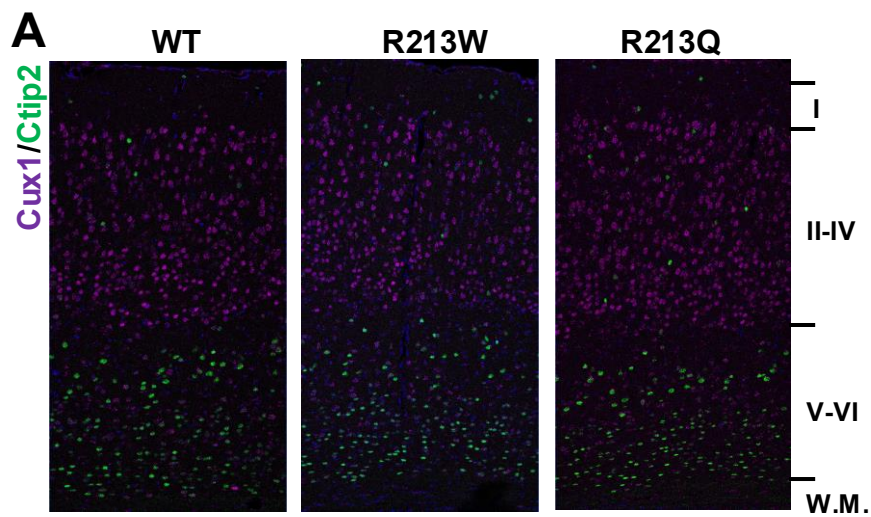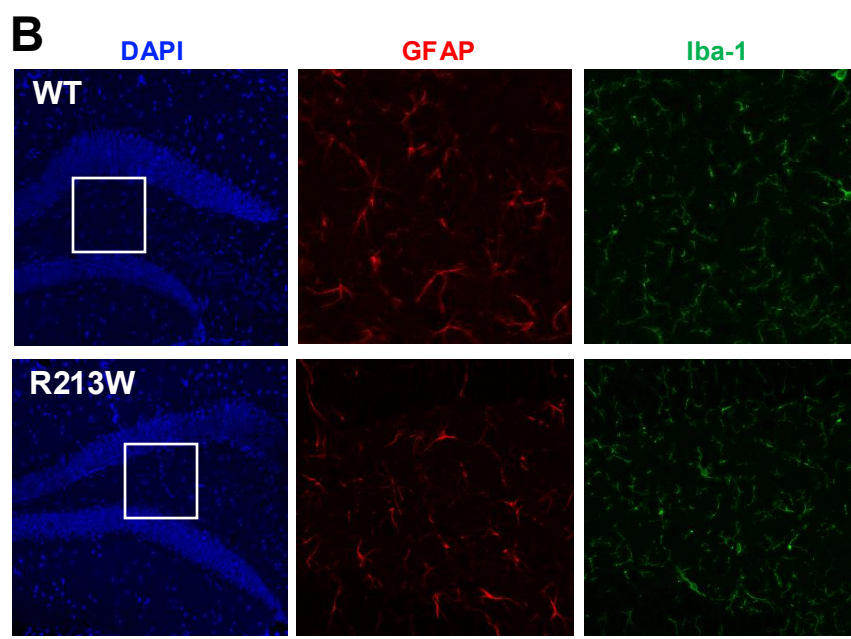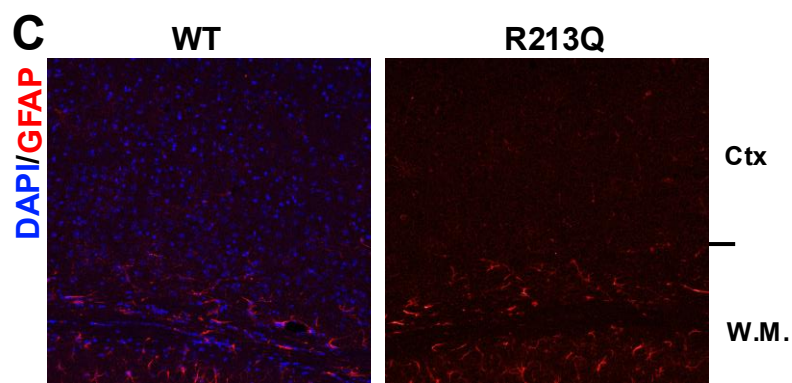

Supplement: Supplementary file 4 — Additional file4 (PDF 206 KB). Supplementary Fig. 4. Morphological analyses of cortical and hippocampal neurons in Kcnq2R213Q/+ mice. (A) Dendritic morphology of cortical neurons. Representative images of Golgi-stained cortical neurons from WT and Kcnq2R213Q/+ mice (upper panel) at P30. Digitally reconstructed dendritic structures are shown in the lower panels. (B, C) Quantification of (A). Violin plots showing the number of dendritic branches (B) and the total basal dendritic length (C) in WT and Kcnq2R213Q/+ mice. n = 4 animals per genotype; 36 and 30 cells for WT and Kcnq2R213Q/+, respectively. No significant differences (ns) were observed. (D) Dendritic spine density in cortical neurons. Representative images of dendritic spines in cortical neurons from WT and Kcnq2R213Q/+ mice. (E) Quantification of (D). Violin plots showing dendritic spine densities (spines per 10 μm) in cortical neurons. n = 4 animals per genotype; 41 and 34 cells for WT and Kcnq2R213Q/+, respectively. No significant differences were detected. (F) Dendritic spine density in hippocampal CA2 (left) and CA3 (right) neurons. Representative images of dendritic spines in neurons from WT and Kcnq2R213Q/+ mice were shown. (G) Quantification of (F). Violin plots showing dendritic spine densities (spine number per 10 μm) in hippocampal CA2 neurons (n = 4 animals per genotype; 30 and 28 cells for WT and Kcnq2R213Q/+, respectively) and CA3 neurons (n = 4 animals per genotype; 27 and 30 cells for WT and Kcnq2R213Q/+, respectively). No significant differences were observed. Scale bar, 50μm (A) and 5μm (D and F). [file 40478_2026_2264_MOESM4_ESM.pdf]

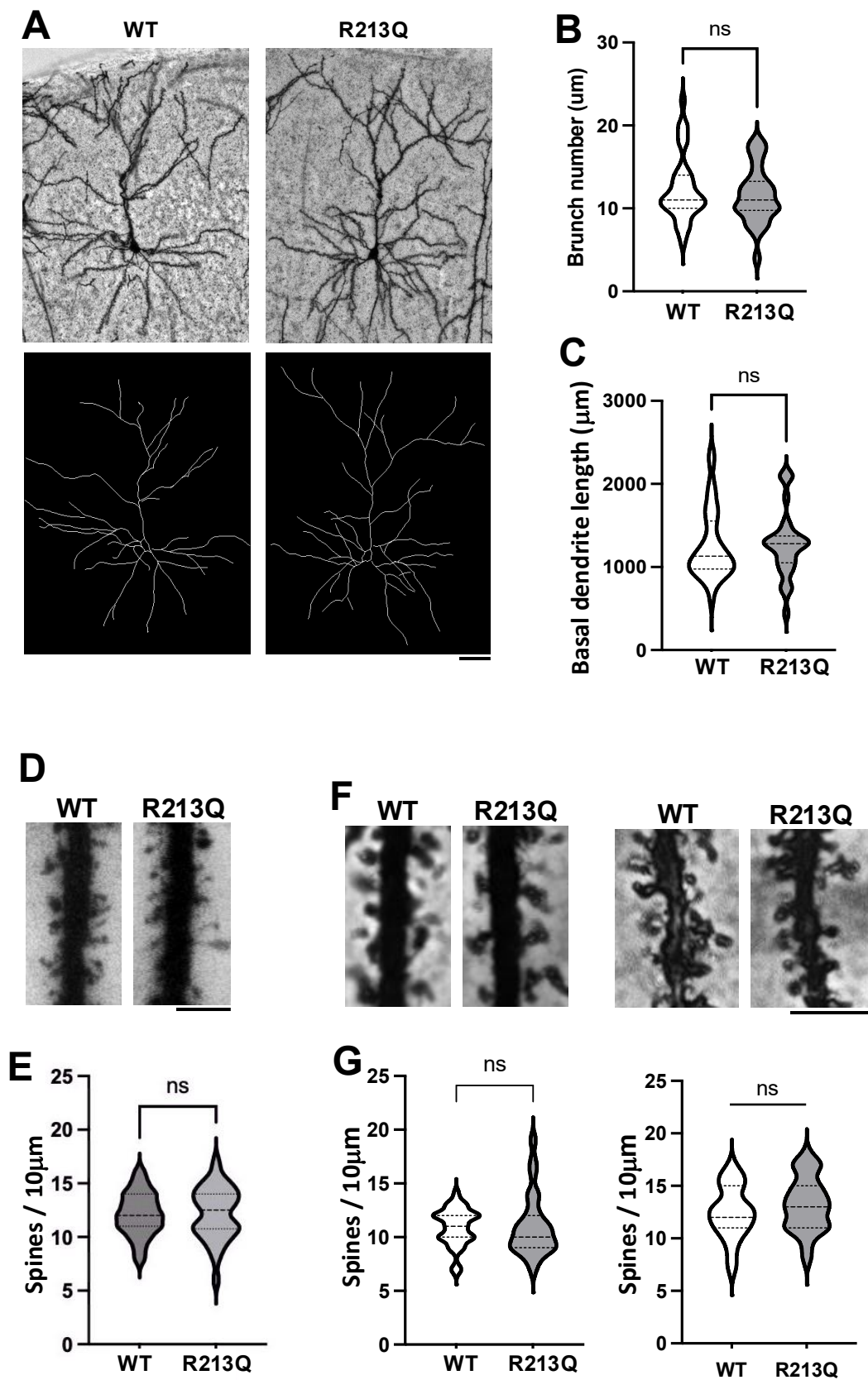

Supplementary Figure 4.

Supplement: Supplementary file 5 — Additional file5 (PDF 354 KB). Supplementary Fig. 5. Synaptic transmission in granule cells of the hippocampal dentate gyrus from Kcnq2R213W/+ and Kcnq2R213Q/+ mice. (A) Representative traces of mEPSCs recorded under the pharmacological conditions described in the “Materials and methods” section. (B) Quantification of mEPSC frequency and amplitude in (A). Recordings were obtained from 6 neurons per genotype (3 slices from 2 mice). (C) Representative traces of mIPSCs recorded under the conditions indicated in “Materials and methods” section. (D) Quantification of mIPSC frequency and amplitude in (C). Recordings were obtained from 6 neurons per genotype (2 slices from 2 mice). The number of mice represents the biological replicates, whereas the number of slices and neurons represents technical replicates. Box plots show the median (horizontal line), interquartile range (box), and full range (whiskers). In the dot plots, different point shapes indicate the individual mice: red circles for mouse #1, squares for mouse #2, and triangles for mouse #3. See “Materials and methods” for details. *P < 0.05; ***P < 0.001 (Tukey-Kramer LSD test). [file 40478_2026_2264_MOESM5_ESM.pdf]

**A**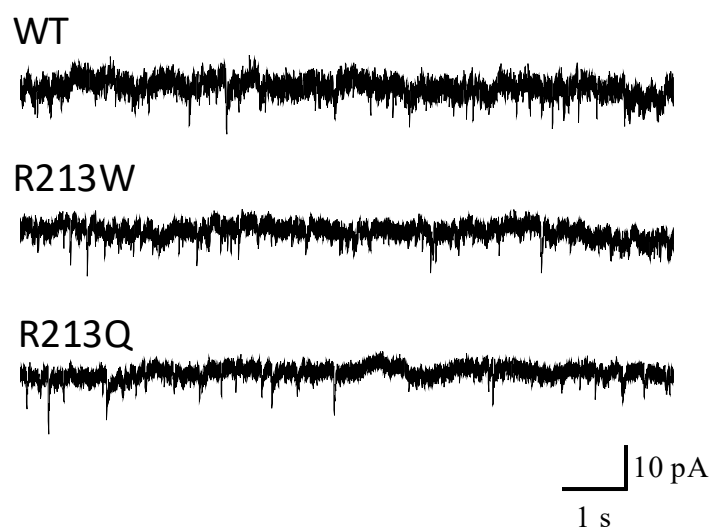**C**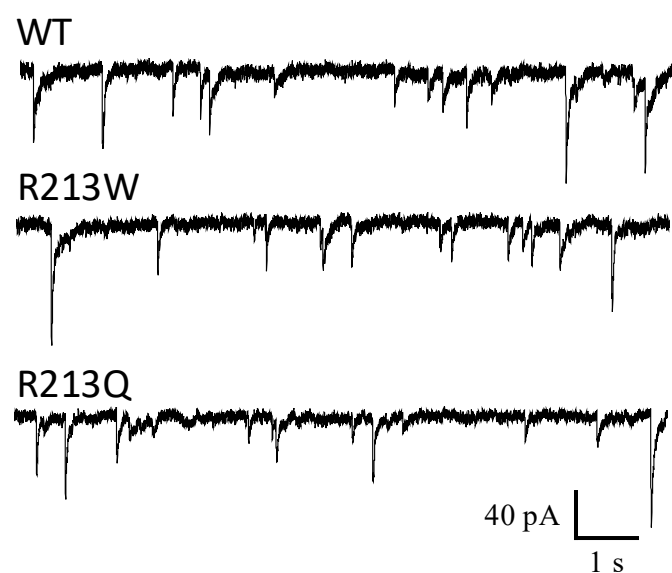**B**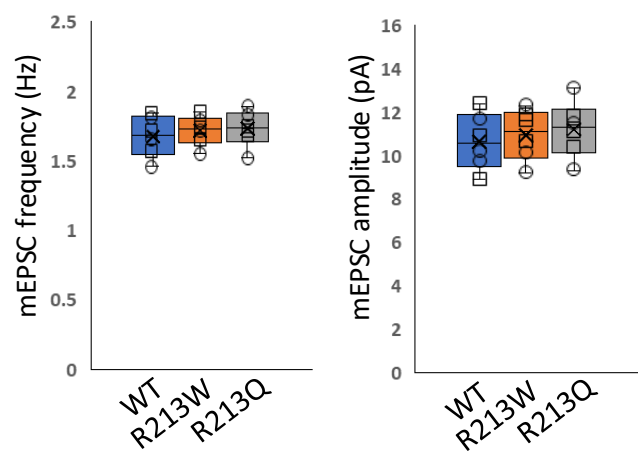**D**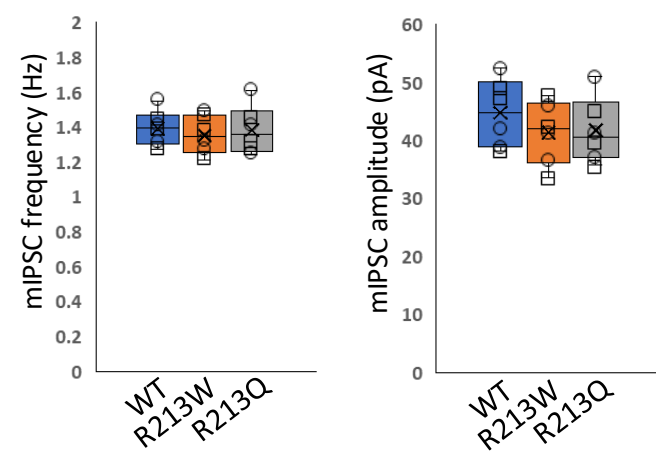

Supplement: Supplementary file 6 — Additional file6 (PDF 220 KB). Supplementary Fig. 6. Synaptic transmission in cortical neurons from Kcnq2R213W/+ and Kcnq2R213Q/+ mice. (A) Representative traces of mEPSCs recorded under the pharmacological conditions described in the “Materials and methods” section. (B) Quantification of mEPSC frequency and amplitude in (A). Recordings were obtained from 6 neurons per genotype (3 slices from 2 mice). (C) Representative traces of mIPSCs recorded under the pharmacological conditions described in the “Materials and methods” section. (D) Quantification of mIPSC frequency and amplitude in (C). Recordings were obtained from 6 neurons per genotype (3 slices from 2 mice). The number of mice represents the biological replicates, whereas the number of slices and neurons represents technical replicates. Box plots show the median (horizontal line), interquartile range (box), and full range (whiskers). In the dot plots, different point shapes indicate the individual mice: circles for mouse #1, squares for mouse #2, and triangles for mouse #3. See “Materials and methods” for details. *P < 0.05; ***P < 0.001 (Tukey-Kramer LSD test). [file 40478_2026_2264_MOESM6_ESM.pdf]

**A**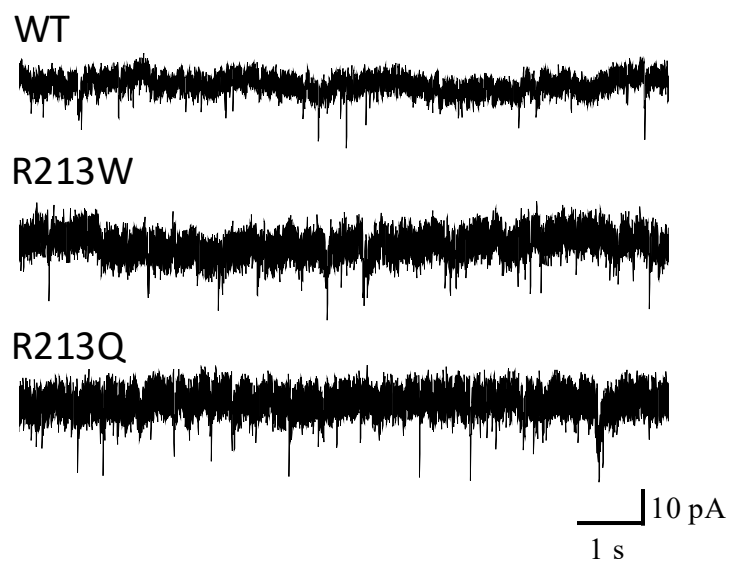**C**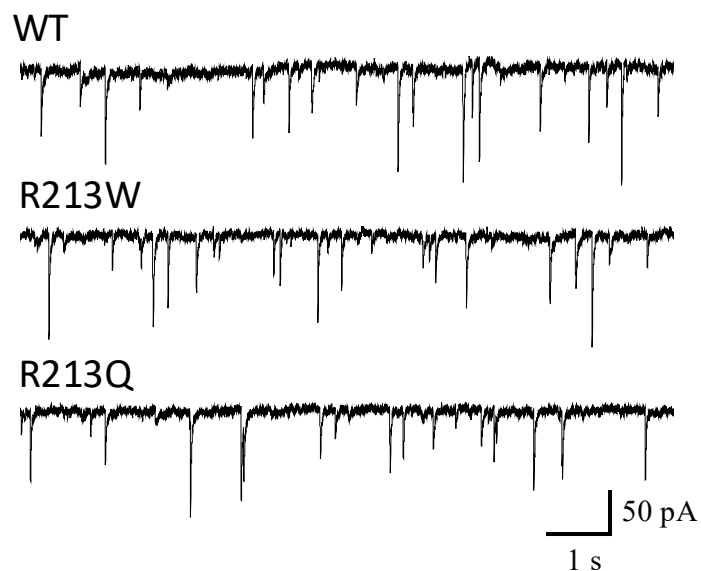**B**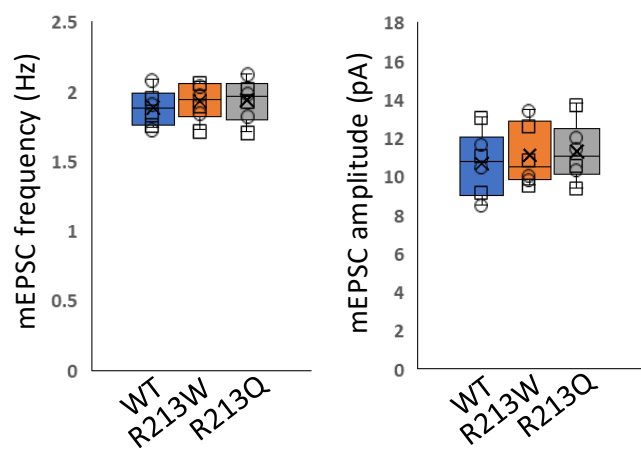**D**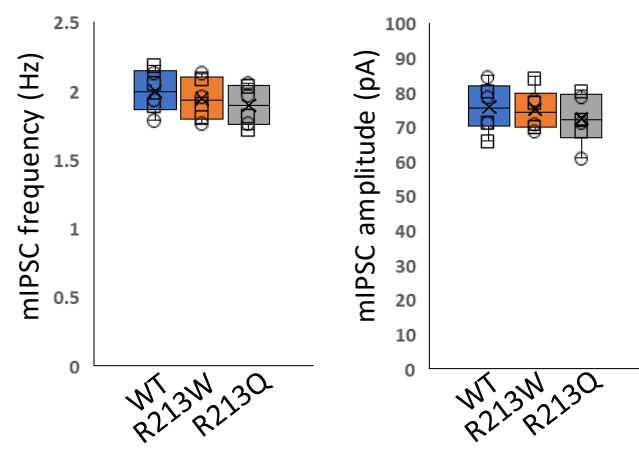

Supplement: Supplementary file 7 — Additional file7 (PDF 218 KB). Supplementary Fig. 7. Quantitative RT-PCR analysis of the differentially expressed genes identified by RNAseq. Relative expression levels of Press41 (A), Mettl27 (B), Egr3 (C), Homer1 (D), Sdf2l1 (E), Hspa5 (F), Slc5a3 (G), and Kcng1 (H) were examined. cDNA was synthesized from total brain RNA using ReverTra Ace reagent (Toyobo, Cat#FSQ-301, Osaka, Japan). qPCR was performed with Thunderbird SYBR qPCR mix (Toyobo, Cat#QPS-201) on a CFX real-time PCR system (Bio-Rad Laboratories, GmbH, Germany) under the following conditions: 95°C for 1 min, followed by 40 cycles of 95°C for 15 s and 60°C for 45 s. Primer sequences were as follows: Press41 (forward: 5’-CTTGAAGAAGTCCCACCGCT-3’, reverse: 5’-GGACGGTCCACTTTTCTGGA-3’), Mettl27 (forward: 5’-GGAACCTCCCACGGTATCAC-3’, reverse: 5’-GTGGGGCTCGGTACTTCAAA-3’), Egr3 (forward: 5’-GATGGCTACAGAGAATGTGATGGA-3’, reverse: 5’- AGTCGAAAGCGAACTTTCCCA-3’), Homer1 (forward: 5’-AGTTTGGCCAATGGGCTGAT-3’, reverse: 5’- GCGACTTCTCCTTTGCAAGC-3’), Sdf2l1 (forward: 5’-CCTCTGTGTTCCTGTCGGTC-3’, reverse: 5’- CCATTGCCTTCCACGTGTTG-3’), Hspa5 (forward: 5’-CGGCTTCCGATAATCAGCCA-3’, reverse: 5’- TCAATCTGGGGAACTCCACG-3’), Slc5a3 (forward: 5’-TGCAGCGAGAATAGCGAAGT-3’, reverse: 5’- ATGACCCATGGAAGCCACTG-3’), Kcng1 (forward: 5’-CCTGGATGAGTTCCCACTGAC-3’, reverse: 5’- GGATGGTGCCAAAAGCTCCG-3’), and GAPDH (forward: 5’-TGATGGGTGTGAACCACGAGAA-3’, reverse: 5’-GGCATGGACTGTGGTCATGAG-3’). Relative expression levels (fold change), normalized to GAPDH and expressed relative to WT, were calculated using the ∆∆Ct method. Differences between R213Q and R213W were assessed by two-tailed unpaired t-test (Prism 10). Press41 (RQ>RW, ***p=0.0001), Mettl27 (RQ<RW, **p=0.0057), Egr3 (RQ>RW, *p=0.0318), Homer1 (RQ>RW, **p=0.0045), Sdf2l1 (RQ>RW, *p=0.0201), Hspa5 (ns, p=0.2126), Slc5a3 (RQ>RW, **p=0.0074), and Kcng1 (RQ>RW, **p=0.0037). [file 40478_2026_2264_MOESM7_ESM.pdf]
