# Supplementary material for: Kcnq2 R213 knock-in mice reveal variant- and region-specific mechanisms underlying self-limited familial neonatal-infantile epilepsy and early infantile developmental and epileptic encephalopathy
Source: Acta Neuropathol Commun. 2026 Feb 25;14:76. doi: 10.1186/s40478-026-02264-4 (PMC13041443; doi:10.1186/s40478-026-02264-4)
Supplement: Supplementary file 2 — Additional file2 (PDF 364 KB). Supplementary Fig. 2. Expression analysis of Kv7.2 and the p.R213W and p.R213Q variants. (A) Developmental expression profile of endogenous Kv7.2 in the mouse cerebral cortex. Western blot analysis (7.5 % gel) was performed using cortical lysates collected at various developmental stages (E17 to P60). GFAP and β-actin were used as an astrocyte marker and loading control, respectively. (B) Expression of Myc-tagged Kcnq2 constructs in COS7 cells. Cells were transfected with pCAG-Myc-Kcnq2 (WT), -Kcnq2-R213W, or -Kcnq2-R213Q (1 μg each). After 48h of incubation, cell lysates were collected and immunoblotted with anti-Myc. (C) Kv7.2 protein levels in Kcnq2R213W/+ and Kcnq2R213Q/+ mice at P34. Western blot analysis with anti-KCNQ2 (10% gel) of cortical lysates from WT, Kcnq2R213W/+, and Kcnq2R213Q/+ mice. β-actin was used to confirm equal protein loading. [file 40478_2026_2264_MOESM2_ESM.pdf]

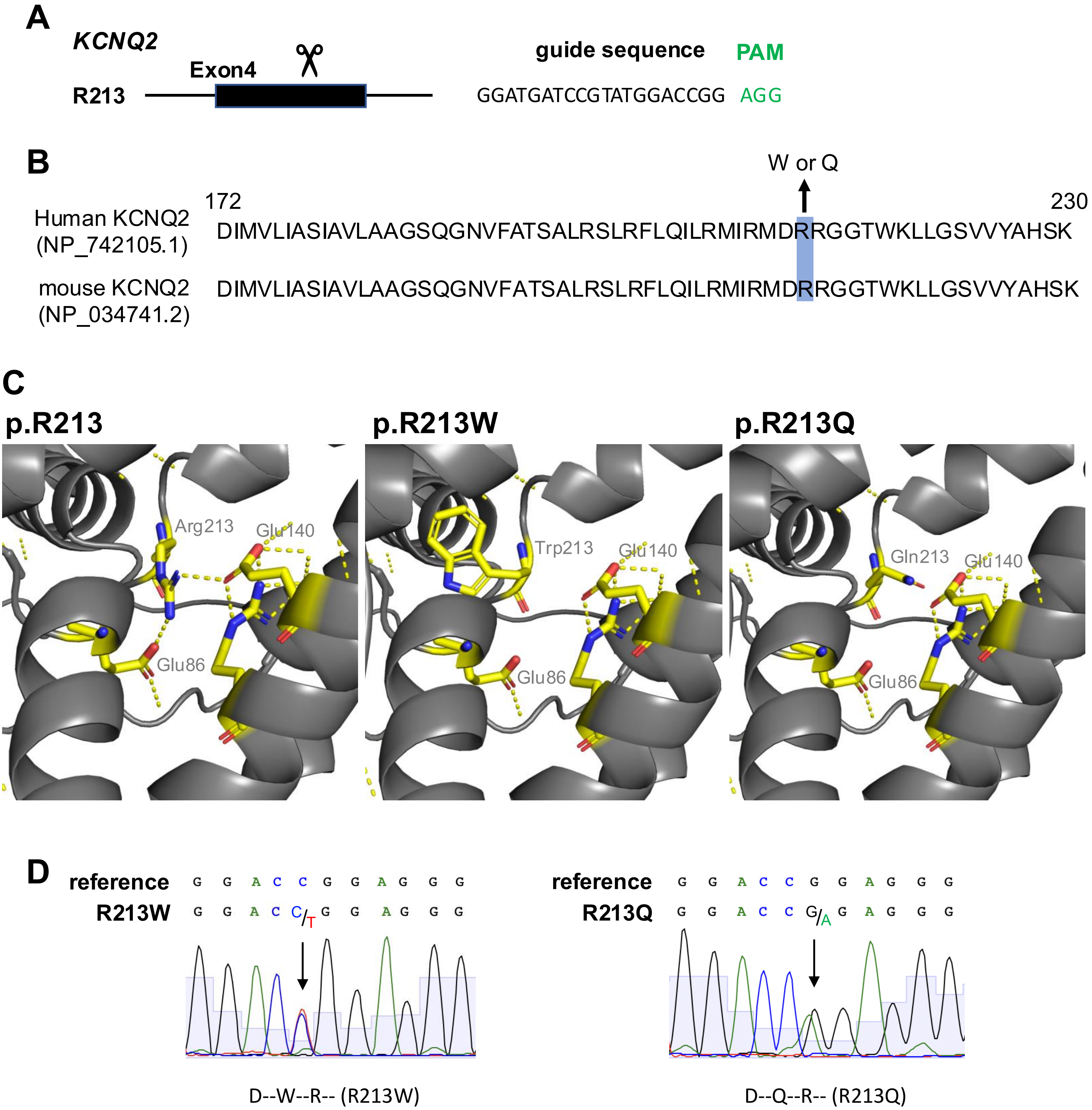

Supplementary Figure 1.
