# Supplementary material for: Kcnq2 R213 knock-in mice reveal variant- and region-specific mechanisms underlying self-limited familial neonatal-infantile epilepsy and early infantile developmental and epileptic encephalopathy
Source: Acta Neuropathol Commun. 2026 Feb 25;14:76. doi: 10.1186/s40478-026-02264-4 (PMC13041443; doi:10.1186/s40478-026-02264-4)
Supplement: Supplementary file 3 — Additional file3 (PPTX 1851 KB). Supplementary Fig. 3. Morphological analyses of the cerebral cortex and hippocampus. (A) Cortical layer organization at P40. Immunostaining was performed using Cux1 (upper-layer marker) and Ctip2 (deep-layer marker), showing normal laminar structure in Kcnq2R213W/+ and Kcnq2R213Q/+ mice. (B) Representative immunofluorescence images of the dentate gyrus from WT and Kcnq2R213W/+ mice at 6–7 months of age. Sections were stained as in Fig. 2A. Boxes in the DAPI panels indicate the areas magnified in the adjacent GFAP and Iba1 panels. Scale bars, 200 μm (left) and 50 μm (right). (C) Distribution of GFAP-positive astrocytes in the cerebral cortex of a 7-month-old Kcnq2R213Q/+ mouse that experienced four seizure episodes. Scale bar, 100 μm. [file 40478_2026_2264_MOESM3_ESM.pptx]

## Slide 1
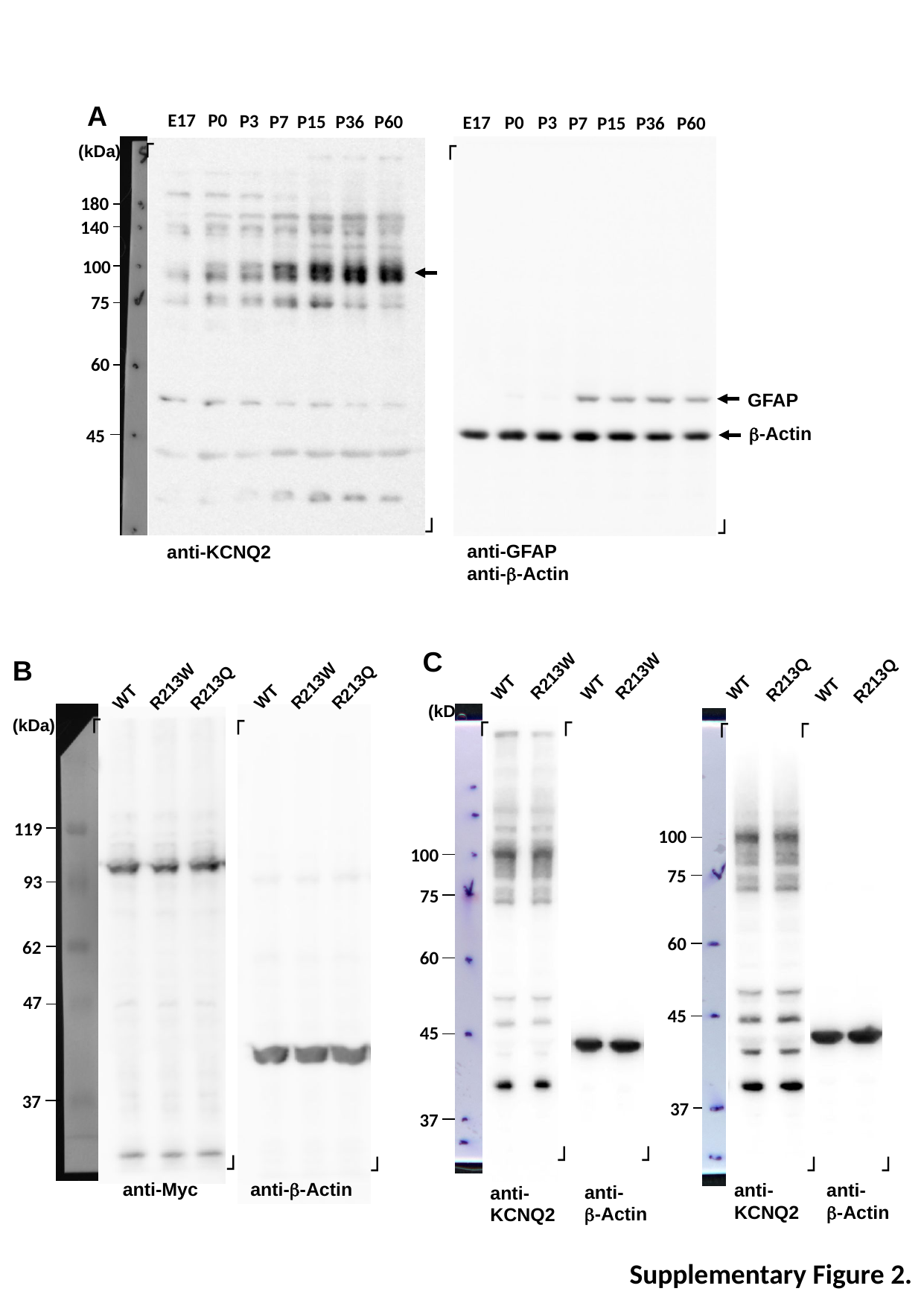

A
E17
P0
P3
P15
P36
P7
P60
E17
P0
P3
P15
P36
P7
P60
「
「
(kDa)
180
140
100
75
60
GFAP
b-Actin
45
「
「
anti-GFAP
anti-b-Actin
anti-KCNQ2
C
B
R213W
R213W
R213Q
R213Q
R213W
R213W
R213Q
WT
WT
R213Q
WT
WT
WT
WT
(kDa)
「
(kDa)
「
「
「
「
「
119
100
100
75
93
75
60
62
60
47
45
45
37
37
37
「
「
「
「
「
「
anti-Myc
anti-b-Actin
anti-KCNQ2
anti-
b-Actin
anti-
b-Actin
anti-KCNQ2
Supplementary Figure 2.
